# Bacterial Communities Associated With Crustose Coralline Algae Are Host‐Specific

**DOI:** 10.1002/mbo3.70213

**Published:** 2026-01-14

**Authors:** Abigail C. Turnlund, Paul A. O'Brien, Laura Rix, Sophie Ferguson, Nadine Boulotte, So Young Jeong, Nicole S. Webster, Guillermo Diaz‐Pulido, Muhammad Abdul Wahab, Miguel Lurgi, Inka Vanwonterghem

**Affiliations:** ^1^ Australian Centre for Ecogenomics The University of Queensland Brisbane Queensland Australia; ^2^ Helmholtz‐Institute for Functional Marine Biodiversity University of Oldenburg Oldenburg Niedersachsen Germany; ^3^ Alfred Wegener Institute Helmholtz‐Centre for Polar and Marine Research (AWI) Bremerhaven Germany; ^4^ Australian Institute of Marine Science Townsville Queensland Australia; ^5^ Coastal and Marine Research Centre, School of Environment and Science, Nathan Campus Griffith University Brisbane Queensland Australia; ^6^ Institute of Marine and Antarctic Studies University of Tasmania Hobart Tasmania Australia; ^7^ Department of Biosciences Swansea University Swansea UK; ^8^ Commonwealth Scientific and Industrial Research Organisation Brisbane Queensland Australia

**Keywords:** coral reefs, Great Barrier Reef, Indo‐Pacific, microbiome, Rhodophyta, symbiosis

## Abstract

Crustose coralline algae (CCA) comprise hundreds of different species and are critical to coral reef growth, structural stability and coral recruitment. Despite their integral role in reef functioning, little is known about the diversity and structure of bacterial communities associated with CCA. We address this knowledge gap by characterising the surface microbial communities of 15 Indo‐Pacific CCA species across eight different families from the Great Barrier Reef, using 16S rRNA amplicon sequencing. CCA microbial community composition was distinct and found to primarily differentiate by algal host species. When looking at the core bacterial communities, divergence across CCA microbiomes was additionally correlated to host phylogeny. CCA from similar light environments and depths also had more similar microbial communities, suggesting the potential role of environmental parameters in influencing microbial community organisation. The fundamental descriptions of CCA bacterial communities for a wide range of Indo‐Pacific species presented here provide essential baseline information to further inform CCA microbial symbiosis research.

## Graphical abstract

Fifteen Indo‐Pacific crustose coralline algae (CCA) species surface microbial communities were characterised with 16S rRNA amplicon sequencing and are distinct. The CCA surface microbiome primarily differentiate by algal host species, but core bacterial communities additionally correlated to host phylogeny.

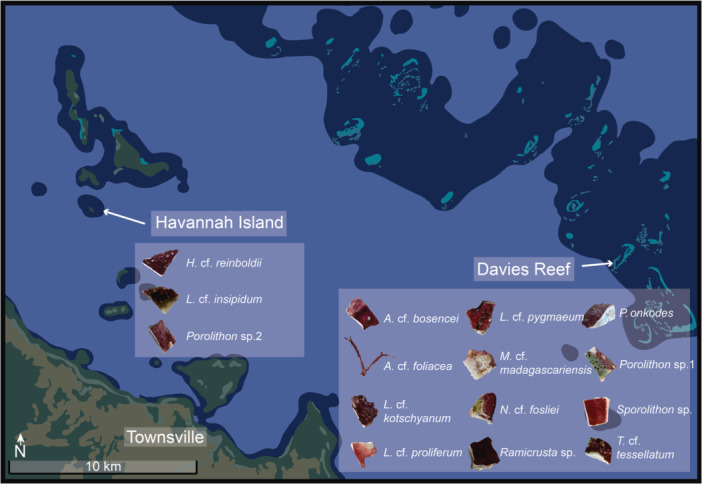

## 1 Introduction

Crustose coralline algae (CCA) are a type of red algae (Corallinophycidae, Rhodophyta) that are critical for tropical coral reef ecosystem functioning by facilitating reef cementation and accretion (Nelson 2009; Silva and Johansen 1986; Schubert et al. 2024). Their rigid high‐magnesium calcite skeleton cements coral rubble and other loose substrates (Littler and Littler 2013; Adey 1998; Steneck 1986; Bergstrom et al. 2020; Quinlan et al. 2019), helping to stabilise reef structures (Rasser and Riegl 2002; Fagerstrom 1987; Scoffin 1992). Calcium carbonate (CaCO_3_) accumulated within CCA can establish coralline beds and ridges on reefs that lessen wave‐impacts on more delicate coral structures and prevent erosion (Littler and Littler 2013; Lewis et al. 2017; Cornwall et al. 2023; Chisholm 2000). This also allows CCA to develop shelf‐like or branching structures that provide safe habitats for a high diversity of marine invertebrates to avoid predation (Littler and Littler 2013; Nelson et al. 2014; Teichert 2014; Riosmena‐Rodríguez et al. 2017). CCA also help maintain healthy reef states by mitigating overgrowth of fleshy algae through sloughing of their upper thallus layer (epithallus) or allelopathic chemicals (Littler and Littler 2013; Keats et al. 1997; Gomez‐Lemos and Diaz‐Pulido 2017). Furthermore, CCA promote reef growth and diversity by inducing settlement for a wide variety of marine invertebrates (Webster et al. 2013b; Johnson and Sutton 1994; Huggett et al. 2006; Siboni et al. 2020; Jorissen et al. 2021; Doll et al. 2023), including corals and abalone (Morse and Morse 1984; Martin and Gattuso 2009; Littler and Littler 2013; Abdul Wahab et al. 2023).

Microbial‐host symbioses are ubiquitous in coral reefs and are crucial for the persistence of fundamental components of these ecosystems. For instance, organisms such as sponges and corals are associated with symbionts involved in a wide‐range of functions, including nitrogen metabolism, nutrient cycling and secondary metabolite production (Taylor et al. 2007; Bourne et al. 2009; Webster and Taylor 2012; Fan et al. 2012; Rosenberg et al. 2007). Symbiotic microbes have also been linked to host health, with dysbiosis observed in diseased green algae (James et al. 2020; Kopprio et al. 2021; Liang et al. 2019), corals (Ng et al. 2015; Zanotti et al. 2021; Meyer et al. 2019; Bourne et al. 2009) and sponges (Luter et al. 2010; Luter and Webster 2022). Despite CCA's importance for reef functioning, there is still very limited information on CCA microbial composition and how microbe‐host interactions influence different aspects of CCA host development, health, and resilience. The few studies that have characterised CCA‐associated microbial communities found that CCA have distinct microbiomes that differ from the surrounding water column (Barott et al. 2011; Cavalcanti et al. 2014; Gefen‐Treves et al. 2021; Siboni et al. 2020; Sneed et al. 2015; Hochart et al. 2024) and broadly consist of *Proteobacteria*, particularly *Alpha‐* and *Gamma‐proteobacteria* (Jorissen et al. 2021; Gefen‐Treves et al. 2021; Quinlan et al. 2019). One study focusing on the CCA *Neogoniolithon* sp. (Gefen‐Treves et al. 2021) revealed that its core microbiome (i.e., a set of microbial taxa consistently found in association with a particular species (Astudillo‐García et al. 2017)) consisted of diverse phyla including *Proteobacteria*, *Bacteroidota*, *Cyanobacteria* and *Actinobacteria* (Gefen‐Treves et al. 2021). Importantly, a number of taxa associated with CCA have been previously linked to anti‐microbial properties (Lera‐Lozano et al. 2025), which may elicit selective pressure and help shape the community composition to select for non‐pathogenic species, symbiotic organisms, or to prevent epibiont settlement (Quinlan et al. 2019). This heterogeneity in microbial composition and function highlights the importance of characterising CCA microbiomes to better understand their persistence and functioning.

Intraspecific differences in microbial diversity and community composition have been attributed to CCA epithallus shedding strategies (i.e., shedding entire sheets of the epithallus at once, shedding in patches, or not at all) and habitat (Jorissen et al. 2021; Sneed et al. 2015). Additionally, microbial composition can change within CCA species in response to variations in the surrounding environment, including seasonal nutrient and temperature fluctuations (Valdespino‐Castillo et al. 2021) and temperature stress (Webster et al. 2011). Furthermore, exposure to low pH has been shown to impact CCA bacterial communities before signs of physiological stress are observed (Webster et al. 2013a). Microbial community variability within CCA species is also hypothesised to reflect host health (Jorissen et al. 2021). Diseases that typically affect reef building coralline algae, including coralline white band syndrome (CWBS) and coralline white patch disease (CWPD), have been associated with distinct pathobiomes (Quéré et al. 2019; Meistertzheim et al. 2017) with lower bacterial diversity observed in diseased CCA (Meistertzheim et al. 2017). Additionally, diseased CCA microbiomes typically show increases in opportunistic taxa. For example, some *Bacteroidota spp*., *Gammaproteobacteria spp*., *Rickettsiales spp*., and *Vibrio tubiashii*, have been found to dominate the microbiome of CWPD‐affected *Neogoniolithon mamillare*, while *Rhodobacterales* were found to dominate CWBS‐affected *N. mamillare* (Meistertzheim et al. 2017). However, these studies typically consist of only a limited number of CCA species and primarily focus on disease status. Since CCA health might be intertwined with their epiphytic communities, an initial understanding of the baseline CCA microbiome is needed before evaluating how stressors like future ocean conditions may affect CCA microbial symbiosis and ultimately CCA health.

Here, we characterised the bacterial communities associated with 15 CCA species from eight families commonly found on the Great Barrier Reef (GBR), enabling us to assess patterns of host‐specificity, identify core microbiomes, and evaluate environmental influences on community composition. This dataset represents the most comprehensive CCA microbiome characterisation to date and provides a valuable baseline for future studies on CCA health and resilience.

## 2 Methods

### 2.1 CCA Sample Collection and Identification

CCA specimens were collected on the Central GBR between the 9th and 20th of October 2021 (GBRMPA Permit G21/45348.1) from Davies Reef (18°49’12.2”S 146°38’39.4”E) (a mid‐shelf low‐turbidity reef), and Havannah Island within the Palm Island Group (18°45’54.8”S 146°31’36.1”E) (inshore islands with fringing reefs) (Figure S1). CCA habitats varied by species and were distributed across reef zones (i.e. reef crest, shallow, mid‐, deep reef) (Table S1). CCA replicates were collected from a single habitat per CCA species. Habitats were described in broad categories relating to reef location, light availability, and sampling depth. Light availability was grouped into three categories (high, moderate, and low) based on sampling depth and habitat (Table S1). Specimens belonged to 15 morphologically diverse species of red algae, including 13 crustose (non‐geniculate) coralline algae, one articulated (geniculate) coralline alga, and one non‐coralline encrusting red alga (Abdul Wahab et al. 2023) (Table S1), collectively hereafter referred to as CCA. Four biological replicates (i.e. samples from different specimens) of each CCA species were collected by SCUBA at least 2 m apart using a hammer and chisel at depths of 1–10 m and placed in new plastic Ziploc bags per CCA species. On return to the surface, samples from each species were held on the ship in separate 70 L flow‐through aquaria per CCA species with unfiltered natural seawater (exchange rate of ~2x/hour) until microbial sampling. A 50% shade cloth was placed over holding tanks to maintain < 100 μmol quanta m^‐2^s^‐^^1^ of ambient/natural light. The CCA samples were first identified visually based on morphological and anatomical characteristics by experts in the Coral Reef Algae Laboratory at Griffith University (Nathan, Australia) (Abdul Wahab et al. 2023). In addition to the CCA samples collected for microbiome analysis, voucher samples for each species were collected for molecular phylogenetic analysis (see section Microbiome analysis: Phylosymbiosis analysis), and species identification was performed by the Coral Reef Algae Laboratory at Griffith University as per Abdul Wahab et al. (2023) and Jeong et al. (2019).

### 2.2 CCA‐Surface Bacterial Communities Sampling and DNA Extraction

For CCA bacterial community characterisation, the thallus surfaces (*n* = 4 per species) were scraped once (1 cm per replicate sample for each species) to collect surface‐associated microbes, except for *A*. cf. *foliacea*, *L*. cf. *kotschyanum* and *Melyvonnea* cf. *madagascariensis*, which have branching morphologies and therefore whole fragments were used. Surfaces were scraped using a sterile scalpel blade and transferred into a cryotube with a probing needle. Scalpel blades and probing needles were washed with 80% ethanol between CCA samples. Cryotubes were frozen in liquid nitrogen on board the vessel, transported to the Australian Institute of Marine Science (AIMS) (Townsville, Australia) and kept at −80°C until being shipped to the Australian Centre for Ecogenomics (ACE) (Brisbane, Australia) for further processing.

CCA bacterial samples were extracted using the lysozyme and proteinase K buffer protocol detailed in Wilson et al. (2002). Genomic DNA was quantified with Qubit 2.0 Fluorometer (Invitrogen) and the Qubit dsDNA HS Assay kit (Invitrogen). DNA quality was measured with the Nanodrop (Thermo Scientific) for 260/280 and 260/230 absorbency ratios and DNA was stored at −20°C prior to sequencing.

### 2.3 DNA Sequencing and Bioinformatic Analysis

DNA amplification was performed using the Earth Microbiome Project primers 515 F ‘GTGYCAGCMGCCGCGGTAA’ and 806 R ‘GGACTACNVGGGTWTCTAAT’ (Caporaso et al. 2011) targeting the V4 region of the 16S rRNA gene. Sequencing was performed at ACE on the Illumina MiSeq platform (2 × 250 bp). Demultiplexed sequences were imported into QIIME2 (version 2022.8) and denoised using the DADA2 plug‐in (Callahan et al. 2016) by merging pair‐end reads and clustering sequences into amplicon sequence variants (ASVs). The forward sequences were truncated to 245 bp, and the reverse sequences were truncated to 183 bp. The first 7 bp of the reverse sequences were removed to eliminate reduced quality bases. Representative ASVs were classified based on the SILVA database (version 138.1, 99_majority taxonomy) (Quast et al. 2012) with the QIIME feature‐classifier classify‐sklearn function for the V4 region. Finally, eukaryote, mitochondria and chloroplast sequences were removed from the final ASV table and ASVs were further filtered to retain those with relative abundances greater than 0.01% in at least one sample with the function filter_taxa() from the OTUTable package (Linz et al. 2017). The retained ASV's hereafter constitute, and are referred to as, the entire CCA bacterial communities. ASVs found in blank samples were removed during the 0.01% filtering step due to low abundances. To maximise the number of sample replicates to be used in our analyses, only samples with less than 10,000 reads (before rarefaction) were removed from the dataset since these samples did not reach a plateau in their rarefaction curves (Figure S2). This included one sample from each *Lithothamnion* cf. *proliferum* (602 reads), *Sporolithon* sp. (1033 reads), and *M*. cf. *madagascariensis* (8388 reads) (Table S2). The relative abundances of CCA‐associated bacterial communities were visualised as stacked bar charts using the package ggplot2 (Wickham 2016), and all analyses were performed in RStudio (Rstudio team 2019) unless otherwise stated.

### 2.4 Bacterial Community Analysis

#### 2.4.1 CCA Bacterial Community Diversity and Structure

Alpha diversity metrics were calculated using the filtered CCA bacterial community dataset rarefied to the depth of the smallest sample (10,567 reads) with the rarefy_even_depth() function from the phyloseq package (Mcmurdie and Holmes 2013). Observed richness index was calculated based on the rarefied data using estimateR(), which estimates species richness from counts per CCA species. An Evenness Index was calculated using the vegan package (Oksanen et al. 2019) by dividing the Shannon Diversity index, calculated with the diversity() function, by the log transformation of the number of unique ASVs, calculated from the specnumber() function. Differences in the observed richness and evenness indices between CCA species were assessed using the non‐parametric Kruskal‐Wallis one‐way analysis of variance with the kruskal.test() function from the base R stats package (Rstudio team 2019).

Multivariate ordination plots using non‐metric multidimensional scaling (nMDS) with Bray‐Curtis dissimilarity were used to visualise the interspecific and intraspecific variability between CCA bacterial communities (i.e. samples) based on non‐rarefied datasets. ASV counts were log transformed and ordination distance matrices were created with the metaMDS() function from the package vegan (Oksanen et al. 2019). Groups within ordination plots were coloured according to CCA species, CCA family, habitat light availability, and collection site to assess the effect of these factors on community similarity and clustering. Visualisation was complemented with quantification of CCA bacterial community dissimilarity by creating a Bray‐Curtis table of the log transformed ASV counts with the vegan function betadiver() using Whittaker's index (Oksanen et al. 2019).

To assess patterns of variability between CCA species, CCA family, habitat light availability, and collection site, the betadisper() function from the vegan package (Oksanen et al. 2019) was used to compare mean centroid distance from log transformed counts at the ASV level, with the bias.adjust set to “True,” given the low sample size. Significance was further determined with an analysis of variance (ANOVA) using the anova() function from the vegan package (Oksanen et al. 2019). To determine whether differences observed between the bacterial communities of various CCA species and environmental variables through multivariate ordination were significant, permutational multivariate analysis of variance (PERMANOVAs) analyses were performed on log transformed counts at the ASV level using CCA species as fixed effects. CCA species were considered as a categorical variable (levels = 15) and fitted independently due to low replication size per CCA species. A Bray‐Curtis dissimilarity distance matrix was calculated with the function vegdist(), while PERMANOVA analysis was calculated using the function adonis2() within the package vegan (Oksanen et al. 2019).

#### 2.4.2 Core Bacterial Community Analysis

Two core bacterial community definitions were used for each CCA species separately by filtering the ASVs to only include those with either 100% or 75% persistence across biological replicates (hereafter referred to as core_100_ and core_75_, respectively) using the filter_taxa() function from OTUTable package (Linz et al. 2017). The filtered ASV tables for all replicates per CCA species were then merged to create a combined core_100_ and a core_75_ CCA bacterial community ASV table. nMDS ordination plots and relative abundance stacked bar charts were used to analyse and visualise the core_100_ and core_75_ CCA bacterial communities as described above for the entire CCA bacterial communities. Additionally, non‐parametric pairwise Wilcoxon tests on taxonomic relative abundance were performed using the pairwise. wilcox. test() function from the base R stats package (Rstudio team 2019).

#### 2.4.3 Phylosymbiosis Analysis

Filtered ASV tables for each species individually were obtained using the filter‐features function with a minimum three sequences and presence in all samples and subsequently grouped into one CCA sample per CCA species with the QIIME2 (version 2022.8) function feature‐table group with the mode mean ceiling. These were then used to create dendrograms of community similarity across species for both core bacterial communities and the entire CCA bacterial community using the diversity beta‐rarefaction function with the Bray Curtis dissimilarity metric. We used the upgma clustering method over 1000 iterations with a sampling depth equal to the sample with the lowest core or entire bacterial community read count (core_100_ = 560, core_75_ = 3072 & entire microbiome =12745) to normalise all CCA species samples for comparison with QIIME2 (version 2022.8). *Ramicrusta* sp. was used as the outgroup for each bacterial community dendrogram, and dendrogram construction was performed with QIIME2 (version 2022.8).

The host phylogenetic tree was created with concatenated DNA barcodes for photosynthetic proteins, *psb*A and *rbc*L (Abdul Wahab et al. 2023) using maximum likelihood analysis with 1000 bootstrap replications from the GTR + G + I model of sequence evolution using RAxMLGUI v1.5 (Abdul Wahab et al. 2023; Stamatakis 2006; Stamatakis et al. 2008; Silvestro and Michalak 2012). Partitioning and evolution models were streamlined with PartitionFinder 2 (Lanfear et al. 2017) and RAxML, respectively. The phylogenetic tree initially contained additional *psb*A and *rbc*L concatenated sequences of other red algae outside this study and were trimmed to only the voucher CCA samples using the ape package (Paradis and Schliep 2018) (Figure S3). *Ramicrusta* sp. was used as an outgroup for the host phylogenetic tree.

Phylosymbiosis analysis was performed by comparing the topology of each bacterial community dendrogram and host phylogenetic tree. For this, phylogenetic tree branch lengths were first removed, then congruence between trees and its significance was calculated based on the normalised Robinson‐Foulds (nRF) metric with 100 permutations (Wickham 2016; Mazel et al. 2018; O'Brien et al. 2020), using the RFmeasures function from Mazel et al. (2018) in R. An additional Mantel test was used to test Pearson correlation between the branch lengths in the bacterial community dendrograms and host phylogenetic tree.

## 3 Results

### 3.1 CCA Bacterial Community Richness and Evenness

**Figure 1:**
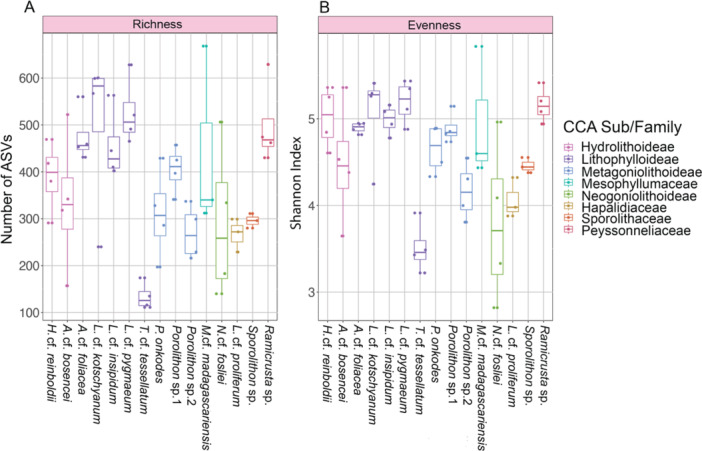
Crustose coralline algae (CCA) bacterial communities differ in richness and evenness. The box plots show the observed amplicon sequence variant (ASV) richness (A) and Shannon evenness (B) for each CCA bacterial community at the ASV level. Each sample is represented as a dot and coloured by its corresponding CCA family/subfamily. Box whiskers highlight the first and third quantiles for each measure per CCA, and the centre line represents the median.

CCA surface bacterial communities statistically differed between CCA species in richness (Kruskal–Wallis H(14) = 34.45, *p* < 0.002) and evenness (Kruskal‐Wallis H(14) = 35.26, *p* < 0.001) (Figure 1; Table S3). In addition, CCA bacterial community diversity varied within CCA families and even between species of the same genus. For instance, within the CCA subfamily Lithophylloideae, *Amphiroa* cf. *foliacea*, *Lithophyllum* cf. *pygmaeum*, *L*. cf. *kotschyanum*, and *L*. cf. *insipidum* had the highest microbial diversity of all observed CCA (mean observed ASV richness: 526 ± 36 standard error, 502 ± 88, 476 ± 29, and 455 ± 37, respectively), whereas *Titanoderma* cf. *tessellatum* was the least microbially diverse (mean observed richness: 134 ± 14). Amongst the three *Porolithon* species, *Porolithon* sp.1 had the most diverse bacterial community (mean observed richness: 405 ± 25) whilst *Porolithon* sp.2 was the least diverse (mean observed richness: 270 ± 29). Furthermore, within‐species variability differed between CCA with *Neogoniolithon* cf. *fosliei, Adeylithon* cf. *bosencei*, *Lithophyllum* cf. *kotschyanum*, and *Melyvonnea* cf. *madagascariensis* having the highest intraspecific variability (difference of the number of ASVs between the highest and lowest sample: 366, 365, 360 and 356, respectively) and *Sporolithon* sp. showing the lowest variability (difference of the number of ASVs between the highest and lowest sample: 31) (Figure 1). For species evenness, *L*. cf. *pygmaeum*, *Ramicrusta* sp., *L*. cf. *kotschyanum*, and *Hydrolithon* cf. *reinboldii* had the highest Shannon Index values (mean Shannon Index: 5.19 ± 0.13, 5.16 ± 0.10, and 5.05 ± 0.27, respectively). Similar to richness, evenness varied highly within *N*. cf. *fosliei*, *A*. cf. *bosencei*, *M*. cf. *madagascariensis*, and *L*. cf. *kotschyanum* samples (difference between the Shannon Index values between the highest and lowest sample: 2.14, 1.71, 1.40 and 1.16, respectively), while *A*. cf. *foliacea* had the lowest variability (difference between the Shannon Index values between the highest and lowest sample: 0.13).

### 3.2 CCA Bacterial Community Composition

**Figure 2:**
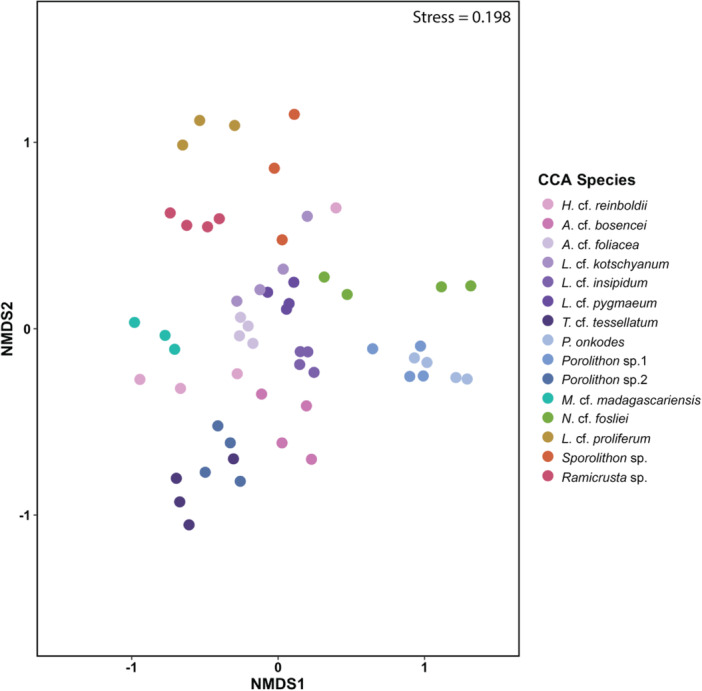
Crustose coralline algae (CCA) bacterial communities differ by algal host. Each point represents a CCA sample and nMDS clusters used Bray‐Curtis distance on log transformed amplicon sequence variant (ASV) counts.

**Figure 3:**
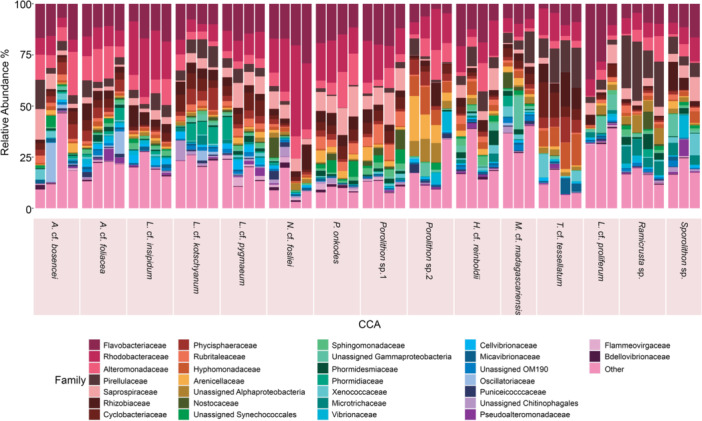
Crustose coralline algae (CCA) bacterial community composition varies between CCA species. The stacked bar plot shows the relative abundance of bacterial families for all CCA samples and grouped per CCA species (*n* = 4). The most abundant 30 families are listed here with less abundant taxa grouped in the ‘Other’ category.

CCA bacterial communities were distinct from each other and grouped by algal host (F = 3.54, *p* < 0.001 PERMANOVA; Figure 2, Figure S4A, Table S4). Furthermore, the dispersion between CCA samples was also significantly different (F = 9.03, *p* < 0.001 ANVOA; Table S5) and therefore may be contributing to differences observed between the different alga hosts. *Proteobacteria* had the highest relative abundance across most CCA species, while *Bacteroidota, Planctomycetota*, *Cyanobacteria*, and *Verrucomicrobiota* were found in each species at varying abundances (Figure S5). At the taxonomic family level, *Flavobacteraiceae*, *Rhodoacteraceae*, and *Alteromondaceae* had the highest abundances amongst most CCA species (Figure 3). Other common taxonomic families that were shared, but varied in relative abundances between CCA species, include *Pirellulaceae*, *Saprospiraceae*, *Rhizobiaceae*, *Cyclobacteraceae*, *Physcisphaeraceae*, *Rubritaleaceae*, *Hyphomonadaceae*, and *Arenicellaceae* (Figure 3).

Overall, the bacterial communities of *Porolithon* sp.1 and *Lithothamnion* cf. *proliferum* were most dissimilar to one another (Figure 2, Table 1). For example, *Porolithon* sp.1 had higher relative abundances of *Phormidiaceae* and *Nostocaceae* than *L*. cf. *proliferum* (Figure 3). Additionally, CCA within the same subfamily had more similar bacterial communities to each other compared to CCA from different subfamilies and orders. An exception being *T*. cf. *tessellatum*, whose bacterial communities most resembled that of *Porolithon* sp.2 with both of these CCA species having the highest relative abundances of *Hyphomonadaceae* compared to the other CCA species (Figure 3, Table 1). Furthermore, when looking at the three *Porolithon* species, differentiation in bacterial communities between species could be observed (Figure 2). Bacterial communities associated with *Porolithon* sp.2 were more dissimilar to *P. onkodes* and *Porolithon* sp.1 compared to any other CCA, while the latter two types had the most similar bacterial communities when comparing all communities against each other (Figure 2, Table 1). For example, *Sapropiraceae* was found in higher relative abundances in *P. onkodes* and *Porolithon* sp.1 than in *Porolithon* sp.2, while *Hyphomonadaceae*, *Arenciellaceae*, and *Vibrionaceae* were found in higher relative abundances in *Porolithon* sp.2 (Figure 3).

**Table 1:** Beta‐diversity based on Bray‐Curtis dissimilarity varied within CCA families, subfamilies, and genotypes. A beta‐diversity score close to 1 (blue – highest value 0.95) indicates high dissimilarity while a beta‐diversity score close to 0 (pink – lowest value 0.57) represent low dissimilarity (high similarity) between bacterial community compositions. CCA labels are coloured by CCA family/subfamily.

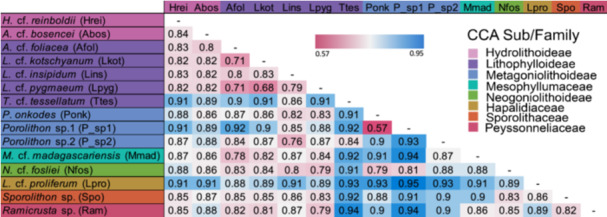

In addition to host‐specific effects, potential impacts of environmental conditions were evaluated as CCA were collected across a range of environments, that is low‐light habitats (crevices and the undersides of coral overhangs), moderate‐light habitats (vertical walls or deeper coral rubble fields) and high‐light habitats (shallow‐reef crests), as well as different sampling sites (Davies Reef and Havannah Island) (Figure S4B, C, Tables S4 and S5). CCA collected from similar light habitats appeared to show similarities in bacterial composition (Figure S4B). For example, CCA found in low‐light habitats (i.e., *L*. cf. *proliferum*, *Ramicrusta* sp., and *Sporolithon* sp.), had lower relative abundances of *Cyclobacteriaceae*, and higher relative abundances of *Microtrichaceae, Woeseiaceae*, and PS1 clade (*Alphaproteobacteria*) (Figure 3; Table S1). Meanwhile, CCA found in moderate‐light habitats (i.e., *H*. cf. *reinboldii*, *M*. cf. *madagascariensis*, and *T*. cf. *tessellatum*) had higher relative abundances of *Xenococcaceae*, while CCA found in high‐light habitats (i.e., *A*. cf. *bosencei*, *A*. cf. *foliacea*, *L*. cf. *insipidium*, *L*. cf. *kotschyanum*, *L*. cf. *pygmaeum*, *N*. cf. *fosliei*, *P. onkodes*, *Porolithon* sp.1, and *Porolithon* sp.2) had higher relative abundances of *Bdellovibrionaceae, Rhizobiaceae*, and *Rhodobacteraceae* (Figure 3; Table S1); However, the coefficient of determination for the source of variation of the bacterial communities was better described by CCA host‐species compared to habitat light conditions or collection site (Table S4).

### 3.3 Structure of the CCA Core Bacterial Community

**Figure 4:**
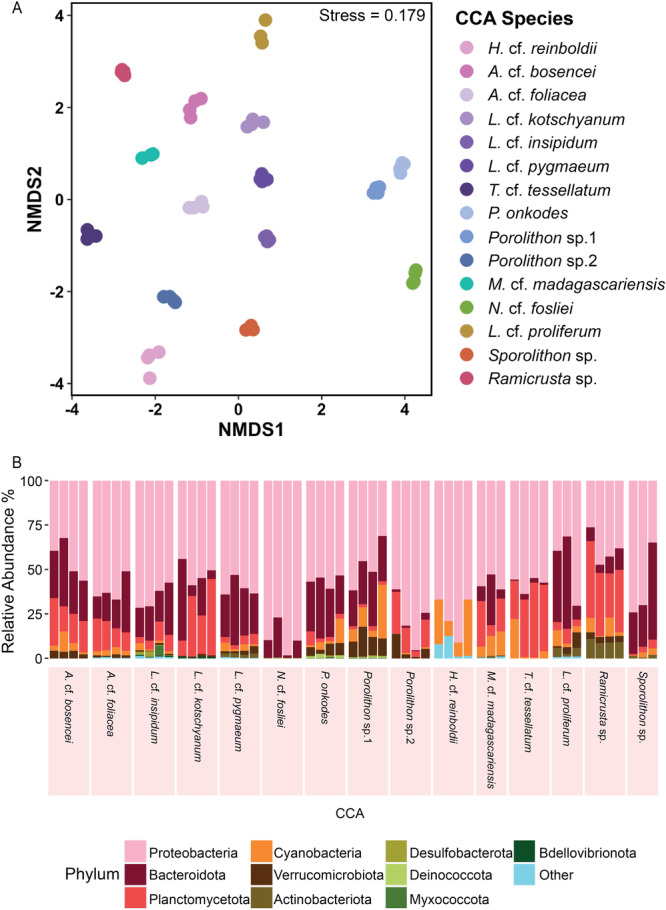
Crustose coralline (CCA) core bacterial communities (100% persistence) are structured by host species. (A) The non‐metric multidimensional scaling (nMDS) plot shows partitioning of samples by CCA species (colour). (B) Stacked bar plot details the relative abundance of each CCA sample at the taxonomic phylum level. The most abundant 15 phyla are listed here with less abundant taxa grouped in the ‘Other’ category.

To further identify host‐specific taxa, individual CCA core bacterial communities were analysed at 75% and 100% persistence (Table S6). Both of these core definitions were considered to capture microbial diversity lost in the stringent 100% persistence definition and compare how this affected downstream phylosymbiosis analysis. Overall, both core bacterial communities grouped by algal host (core_100_: PERMANOVA: F = 168.3, *p* < 0.001; Figure 4A; core_75_: PERMANOVA: F = 35.91, *p* < 0.001; Figure S6) and these groupings were stronger compared to the entire microbial communities groupings by host (Figure 2). Additionally, there were clear differences in bacterial relative abundances that were observed at the phylum level amongst the different CCA species (Figure 4B, Figure S6). Although *Proteobacteria* had the highest overall relative abundance for all CCA species, its abundance and that of other phyla, like *Bacteroidota, Cyanobacteria*, and *Planctomycetota*, varied between CCA species, while other phyla were exclusive to specific CCA (Figure 4B, Figure S6). For instance, *N*. cf. *fosliei* core_100_ bacterial community showed the lowest richness overall, and only included ASVs found in *Proteobacteria, Bacteroidota*, and *Desulfobacteria* (Figure 4B, Table S7). Furthermore, *H*. cf. *reinboldii* was the only CCA to not have *Bacteroidota* included in the core_100_ bacterial community (Figure 4B). *Deinococcota* was unique to *P. onkodes* and *Porolithon* sp.1, while *Myxococcota* and *Bdellovibrionota* were present in *P. onkodes* and *Porolithon* sp.1, but not *Porolithon* sp.2 (Figure 4B, Table S7). Moreover, *N*. cf. *fosliei* and *H*. cf. *reinboldii* had no *Planctomycetota* present in the core_100_ bacterial community, while *L*. cf. *kotschyanum* and *N*. cf. *fosliei* had no *Cyanobacteria* present (Figure 4B, Table S7). At lower taxonomic levels, differences in relative abundance and presence/absence of certain microbial taxa became more pronounced (Figures S7 and S8). For example, *N*. cf. *fosliei* was unique as it had the highest relative abundance of *Rhodobacteraceae* (69.97% ± 5.30%) in the core_100_ bacterial community, whereas *H*. cf. *reinboldii* had the highest relative abundance of *Pseudoalteromonadaceae* (29.44% ± 6.89%) (Figure S7, Table S8). *Ramicrusta* sp. and *L*. cf. *proliferum* also had greater relative abundance of *Microtrichaceae* (7.64% ± 0.51% and 3.75% ± 1.20%, respectively) than other CCA in their core_100_ bacterial communities, while *Porolithon* sp.2 had the highest relative abundance of *Puniceicoccaceae* (5.50% ± 2.91%) (Figure S7, Table S8). Lastly, *Alteromonadaceae* was absent in *M*. cf. *madagascariensis* core_100_ bacterial community, despite being present in all other CCA cores (Figure S7, Table S8).

Similar microorganism abundance patterns were also observed in the core_75_ bacterial communities (Figure S8). Interestingly, *L*. cf. *proliferum*, *Sporolithon sp*., and *M*. cf. *madagascariensis* had the same core bacterial community composition in both the 75% and 100% filtering cut‐offs at the ASV level (Table S6). For all other CCA species, increasing the cut‐off to 100% resulted in a loss of taxa of up to 77% at the ASV level. At the phylum level, the core_75_ and core_100_ CCA bacterial communities mostly differed in abundance, although a few CCA lost phyla in the core_100_ bacterial communities that were present in the core_75_ communities. For example, *Cyanobacteria* was present in the core_75_ bacterial community of *H*. cf. *reinboldii*, *N*. cf. *fosliei*, and *L*. cf. *kotschyanum*, but was not detected in their core_100_ bacterial communities (Figure 4 and Figure S6). Furthermore, *Planctomycetes* was also not detected in *H*. cf. *reinboldii* and *N*. cf. *fosliei* core_100_ bacterial communities but was present in the core_75_ bacterial community (Figure 4 and Figure S6).

### 3.4 Phylosymbiosis in the CCA Core Bacterial Community

**Figure 5:**
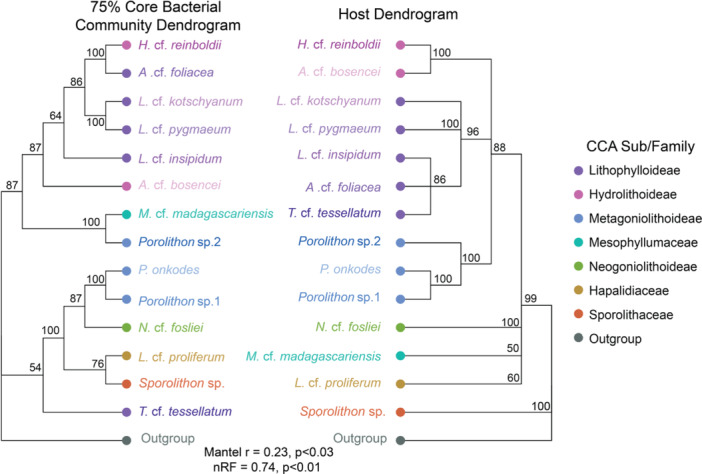
Deviations between CCA species' 75% core bacterial community compositions mirror changes in host phylogeny. Nodes (branch tips) and branch labels are coloured by CCA sub/family and branch labels depict CCA species names. *Ramicrusta* sp. is a non‐CCA species that was used as an outgroup for the host and bacterial community dendrograms.

Phylosymbiosis analysis was performed for the entire, core_75_ and core_100_ bacterial communities (Figures S9, 5, and S10). Here, we focus on the core_75_ (Figure 5) and the entire bacterial community results (Figure S9), since the core_100_ bacterial communities resulted in lower bootstrap scores due to lower taxa present as a result of the more stringent cut‐off (Figure S10). The core_75_ bacterial communities showed host specificity and weak patterns of phylosymbiosis, with CCA from the same subfamily mostly grouping together (Mantel correlation = 0.23, *p* < 0.03; topological congruency nRF = 0.74, *p* < 0.01; Figure 5). For example, all members of the subfamily Lithophylloideae grouped together (except *T*. cf. *tessellatum*), while also grouping with *H*. cf. *reinboldii* and *A*. cf. *bosencei* of the Hydrolithoideae family in the bacterial dendrogram (Figure 5), supporting previous observations based on the dissimilarity matrix and ordinations. Furthermore, two members of the subfamily Metagoniolithoideae remained in a single clade within the bacterial dendrogram (*P. onkodes* and *Porolithon* sp.1), yet *Porolithon* sp.2 grouped with *M*. cf. *madagascariensis*. Further deviations from the CCA phylogenetic tree groupings in the bacterial dendrogram were observed for CCA of the family Hydrolitholideae, which grouped together based on phylogeny, but this was not congruent with the bacterial dendrogram. On the other hand, the placement of CCA families consisting of only a single CCA representative was not conserved in the bacterial dendrogram as they formed clades with CCA of similar bacterial community compositions (Figure 5), likely due to limited representation. Similar trends were also found in the entire bacterial dendrogram (Mantel correlation = 0.30, *p* < 0.07; topological congruency nRF = 0.42, *p* < 0.001; Figure S9). One deviation from the core_75_ bacterial microbial tree compared to the entire bacterial community is that *Porolithon* sp.2 grouped with *T*. cf. *tessellatum* instead of *M*. cf. *madagascariensis* (Figure S9).

## 4 Discussion

### 4.1 CCA Bacterial Communities Are Distinct and Differ in Diversity and Composition

The surface bacterial communities of 15 CCA species were comprehensively characterised, many for the first time, in order to provide a baseline understanding of these host‐microbe associations and generate new hypotheses for future CCA‐microbe symbiosis exploration. Previous research on a limited number of CCA has established that surface‐associated microbiomes have distinct communities that differ between CCA species, even within genera (Jorissen et al. 2021; Sneed et al. 2015; Siboni et al. 2020). This study expands on those results by characterising the bacterial communities of 15 CCA species widely found across the GBR and highlighting that variability in community composition, richness, and evenness amongst CCA surface bacterial communities is primarily driven by host identity despite weak correlations with host phylogeny.

CCA bacterial communities varied in diversity across species. For example, *Titanoderma* cf. *tessellatum* communities had the lowest mean richness and evenness, while *Lithophyllum* cf. *kotschyanum* had the highest mean richness and evenness. Differences in bacterial community diversity could reflect differences in functional potential amongst microbial symbionts and their algae host. This pattern has been observed in other marine benthic organisms. For example, sponge species with low microbial diversity have less functional diversity compared to sponges with high microbial diversity (Lesser et al. 2022). Furthermore, differences in microbial diversity have been shown to be reflected in microbial symbiont‐host processes as high microbial diversity sponges have higher nitrification rates than low microbial diversity sponges (Ribes et al. 2012). Therefore, differences in microbial diversity between CCA like *T. tessellatum* and *L. kotschyanum* may reflect functional specialisation. Additionally, *Neogoniolithon* cf. *fosliei*, *Hydrolithon* cf. *reinboldii*, *Porolithon onkodes, Porolithon* sp.1, and *Porolithon* sp.2 showed high interspecific variation in richness and evenness. All of these species are observed to shed their epithelial cells to prevent unwanted overgrowth, while other CCA species shed less frequently (Harrington et al. 2004). Differences in microbial richness and evenness amongst replicates for these CCA may be attributed to different stages of shedding amongst the individual samples, therefore causing different successional stages of the microbial communities (Sneed et al. 2015; Bengtsson et al. 2012).

Several phyla were shared across all CCA species, including *Proteobacteria, Bacteroidetes* and *Acidobacteria*. These phyla are commonly associated with algae (Ismail et al. 2018; Hollants et al. 2013; Singh and Reddy 2014) and CCA microbiomes (Brodie et al. 2016; Sneed et al. 2015; Gefen‐Treves et al. 2021; Siboni et al. 2020). Taxa belonging to these groups have been hypothesised to perform functions in symbiosis with the host relating to carbon metabolism (Gefen‐Treves et al. 2021; Quinlan et al. 2019) or CCA health (Harder et al. 2012; Egan et al. 2013; Singh and Reddy 2014; Brodie et al. 2016) due to their organic carbon utilisation and antimicrobial properties. Other taxa that are shared amongst CCA species, but vary in abundance depending on the CCA host, include *Saprospiraceae, Rhodobacteraceae, Cyclobacteriaceae* and *Hyphomonadaceae*. These taxa have been associated with macroalgae epiphytic microbiomes (Briggs et al. 2021; Twist et al. 2024; Lu et al. 2022; Huggett et al. 2018; Paix et al. 2019) and change in abundance depending on the season (Paix et al. 2019) and temperature (Huggett et al. 2018). Other studies have shown that CCA microbial communities change when environmental conditions such as pH and temperature are altered (Webster et al. 2011; Webster et al. 2013b). We reaffirmed that CCA surface bacterial communities are distinct between different species, but additional investigation is needed to determine whether key differences between species are a result of host specialisation.

### 4.2 The CCA Core Bacterial Community Is Species‐Specific

When examining the CCA core_75_ and core_100_ bacterial communities (i.e. taxa that were found in 75% and 100% of biological replicate samples per CCA, respectively) the most abundant phyla shared across all CCA species were *Proteobacteria, Bacteroidetes*, and *Acidobacteria*. Most of the patterns observed in the core_100_ bacterial communities relating to presence/absence of individual taxa and relative abundances were also observed in the core_75_ bacterial communities, highlighting the robustness of the core bacterial community definition in our dataset. Interestingly, CCA core bacterial communities differed from each other through the presence and absence of certain taxonomic groups. For example, *Melyvonnea* cf. *madagascariensis* was the only CCA that did not contain *Alteromonadaceae* in either core bacterial community definition, which includes members that are suspected opportunistic pathogens involved in algal bleaching (Yang et al. 2021; Kumar et al. 2016). Furthermore, the core_75_ and core_100_ bacterial communities of different *Porolithon spp*. genotypes, sp.1 and sp.2, featured no *Firmicutes* and low relative abundances of *Actinobacteria*, similar to previously characterised *P. onkodes* core microbial communities (Yang et al. 2021). Recent studies have postulated that algae‐surface microbiomes are shaped by the type of algal exudates they provide to cater to the metabolism of microorganisms (Egan et al. 2013; Burke et al. 2011; Goecke et al. 2010; Cirri and Pohnert 2019; Selvarajan et al. 2019). Therefore, alga host‐specific differences, like nutrient availability on the thallus, could be responsible for the differences in bacterial community composition amongst the CCA species observed here. For example, *H. reinboldii* and *P. onkodes* were observed to have different microbial composition, evenness, and exudate quantities, with *H. reinboldii* exudates being observed in higher quantities (Quinlan et al. 2019). While the relationship between CCA exudates and microbial composition is unclear, the greater bacterial community evenness in *H*. cf. *reinboldii* compared to the three *Porolithon* genotypes also in this study suggests the potential role of nutrient availability in shaping CCA microbiomes should be further explored.

Since CCA core bacterial communities were found to be host‐specific in our analyses, we investigated whether (dis)similarity between CCA microbial communities reflected host phylogeny (Brooks et al. 2016; Brucker and Bordenstein 2013; Lim and Bordenstein 2020; O'Brien et al. 2020). In general, CCA within host phylogenetic clades loosely grouped together based on their bacterial community composition, suggesting the potential for shared evolutionary histories between hosts and microbes. This was most evident in CCA of the subfamily Lithophylloideae, however, the number of species per CCA subfamily sampled differed, and some families/subfamilies lacked species diversity (e.g. Hapalidiaceae and Sporolithaceae). Although there were patterns consistent with phylosymbiosis in both the CCA core_75_ and entire bacterial communities, incongruencies with host phylogeny were observed in the separation of *Porolithon* sp.2 and *T*. cf. *tessellatum* core bacterial communities from other CCA in the Metagoniolithoideae and Lithophylloideae, respectively. Differences in the core_75_ bacterial communities between CCA species of the Metagoniolithoideae include the absence of *Desulfobacterota* in *Porolithon* sp.2, which could relate to host health, as higher abundances of *Desulfobacterota* were found in healthy *P. onkodes* individuals compared to bleached ones in previous studies (Yang et al. 2021). In our study, however, all *Porolithon* individual samples, across genotypes, looked visually healthy when collected. *Porolithon* sp.2 also had the highest relative abundances of *Vibrionaceae*. Given that some members of this family are pathogens while others possess antimicrobial properties, this could further alter the microbial composition (Goecke et al. 2010; Kanagasabhapathy et al. 2006; Hollants et al. 2013) across the *Porolithon* species. For example, the introduction of *Vibrio* species to coral *Montastraea cavernosa* microbiomes triggered dysbiosis with an influx of opportunistic bacteria (Welsh et al. 2017). Furthermore, *T*. cf. *tessellatum* grouped outside the Lithophylloideae clades, which differentiated from other CCA species within the same family with high relative abundances of *Kiloniellaceae*, a family with members involved in nitrogen cycling (Pushpakumara et al. 2023; Imhoff and Wiese 2014). *Kiloniellaceae* is a hypothesised algal symbiont (Pushpakumara et al. 2023) that may utilise compounds produced by *T*. cf. *tessellatum* as a nitrogen source.

### 4.3 Environmental Conditions Potentially Influence CCA Microbiome Composition

In this study, we observed that CCA species found in low‐ and high‐light environments showed differences in relative abundance of specific groups, with *Rhodobacteraceae* being present at higher relative abundance in CCA collected from high‐light environments, which mainly includes *A*. cf. *bosencei*, *A*. cf. *foliacea*, *L*. cf. *insipidium*, *L*. cf. *kotschyanum*, *L*. cf. *pygmaeum*, *N*, cf. *fosliei* and *P. onkodes*. Many members of the *Rhodobacteraceae* are opportunistic microorganisms (Fang et al. 2022) involved in organic matter degradation (Gómez‐Consarnau et al. 2019; Pohlner et al. 2019; Fang et al. 2022) and have also been identified in seagrass epiphytic microbiomes growing in high‐light and high wave action habitats (Rotini et al. 2017; Szitenberg et al. 2022), similar to conditions on the reef crest zones sampled here. Other taxa specific to collection habitats include *Microtrichaceae* and *Woeseiaceae*, which were dominant in CCA collected in low‐light environments, like *L*. cf. *proliferum*, *Ramicrusta sp*., and *Sporolithon sp*. These taxonomic families are potentially involved in ammonia oxidation (Szitenberg et al. 2022), and the sulphur and nitrogen cycles (Mußmann et al. 2017; Fang et al. 2022; Chen et al. 2018; Satoh et al. 2006), respectively. Microorganisms associated with ammonia oxidation can be sensitive to light via photoinhibition (Merbt et al. 2017) and can be distributed amongst a gradient depending on light and depth in oceans (Lu et al. 2020; Ward 1985; Church et al. 2010). Therefore, higher abundances of *Microtrichaceae* on CCA found in low‐light than CCA found in high‐light environments may be reflective of *Microtrichaceae* environmental preferences, and thereby ability for colonisation, in dark and light conditions. Further experimentation would be needed to confirm light sensitivity and distribution of *Microtrichaceae* members, and whether surrounding seawater microbiome composition directly influences CCA microbiomes. In addition, CCA found in moderate‐light habitats, such as *H*. cf. *reinboldii*, *M*. cf. *madagascariensis*, and *T*. cf. *tessellatum*, possessed higher relative abundances of *Xenococcaceae*, similar to results from a study that characterised and compared mangrove sediment microbial communities between lower and upper tidal zones, which found an increase of *Xenococcaceae* abundance in lower‐light lower tidal zone conditions (Zhu et al. 2018). *Xenococcaceae* is a cyanobacterium that can tolerate low irradiance (Mulec et al. 2008; Albertano et al. 2000) and higher abundances of *Xenococcaceae* on CCA found in moderate‐light conditions may be the result of niche partitioning within Cyanobacteria. Previous studies have also found that irradiance tolerance levels are species‐specific within Cyanobacteria and can result in vertical stratification of species presence within the water column (Eigemann et al. 2018; Olli et al. 2015). Despite these qualitative observations, further investigation into the impact of habitat and light conditions is required since individual CCA species were only collected from a single site, that is not across an environmental gradient, and light conditions were not explicitly measured only broadly characterised based on habitat and depth. While our study provides a valuable baseline leading to multiple interesting hypotheses, future studies should expand on this research and include in‐situ measurements of a variety of environmental parameters that could shape epiphytic microbial communities, including pH (Webster et al. 2013a), temperature (Webster et al. 2011), nutrient concentrations (Szitenberg et al. 2022), *p*CO_2_ levels (Webster et al. 2013a), light (Jorissen et al. 2021), depth (Brodie et al. 2016), and hydrodynamics (Jorissen et al. 2021; Proia et al. 2012; Ylla et al. 2012; Misic and Covazzi Harriague 2019). Additionally, sampling the microbial communities of a single species collected across different habitats and, for widely distributed species, geographic ranges (i.e. *P. onkodes*, *N. fosliei*, and *H. reinboldii* (Dean et al. 2015)), should be explored to further establish persistence of species‐specificity of the CCA bacterial communities described here over space, time, and/or seasons.

### 4.4 Concluding Remarks

This study represents the largest characterisation of CCA bacterial communities to date and identifies core microbial taxa for 15 CCA species that are ecologically relevant to the GBR. Our results show that CCA bacterial community composition and diversity are host‐specific, despite showing only weak patterns of phylosymbiosis. Host‐specificity may suggest an important role for the microbial communities in nutrient cycling and maintaining CCA health, similar to other important coral reef organisms like corals and sponges. Future studies that characterise the functional capability of different CCA bacterial communities would provide further evidence for this. Furthermore, the surrounding environmental conditions of cryptic and exposed CCA habitats should be further probed to evaluate the influence of the environment shaping CCA bacterial communities. As CCA play a key role in building and maintaining healthy coral reef ecosystems, the CCA bacterial community descriptions provided here represent important baseline information for understanding CCA‐microbiome interactions and symbiotic relationships that influence CCA health and ecosystem functions.

## Author Contributions

**Abigail C. Turnlund:** conceptualisation (supporting), writing – original draft (lead), formal analysis (lead), writing – review and editing (equal), **Paul A. O'Brien:** conceptualisation (supporting), formal analysis (supporting), writing – review and editing (equal), **Laura Rix:** conceptualisation (supporting), formal analysis (supporting), writing – review and editing (equal), **Sophie Ferguson:** investigation (supporting), writing – review and editing (equal), **Nadine Boulotte:** investigation (supporting), writing – review and editing (equal), **So Young Jeong:** investigation (supporting), writing – review and editing (equal), **Nicole Webster:** conceptualisation (supporting), formal analysis (supporting), writing – review and editing (equal), **Guillermo Diaz‐Pulido:** investigation (lead), writing – review and editing (equal), **Muhammad Abdul Wahab:** investigation (lead), writing – review and editing (equal), **Miguel Lurgi:** conceptualisation (supporting), formal analysis (supporting), writing – review and editing (equal), **and Inka Vanwonterghem:** conceptualisation (lead), formal analysis (supporting), writing – review and editing (equal).

## Ethics Statement

The authors have nothing to report.

## Conflicts of Interest

The authors declare no conflicts of interest.

## Supporting information


**Figure S1:** Map of crustose coralline algae (CCA) collection sites across Havannah Island and Davies Reef, central Great Barrier Reef, off the coast of Townsville, Australia. Davies Reef represents a low‐turbidity, pristine mid‐shelf reef and Havannah Island represents a fringing reef on an inshore island. **Figure S2:** CCA samples rarefaction curve. Samples that were removed from low read count are labelled with purple stars, and the sample used as the cutoff to rarify the dataset for alpha diversity analysis is labelled with a green star. **Figure S3:** Crustose coralline algae (CCA) phylogenetic tree based on Maximum Likelihood trimmed to the CCA vouchers used in this study of combined *psb*A and *rbc*L concatenated sequences. CCA are shaded in their shared sub/family and values at the beginning of branches represent maximum likelihood bootstrap values (%). Reference numbers located at the beginning of each CCA name are the herbarium numbers used in the personal collection of Diaz‐Pulido at Griffith University, Brisbane, Australia. **Figure S4:** Crustose coralline algae (CCA) bacterial communities vary depending on (A) CCA family, (B) CCA habitat light exposure, and (C) collection site. Each point represents a CCA sample and nMDS clusters used Bray‐Curtis distance on log transformed amplicon sequence variant (ASV) counts. **Figure S5:** Crustose coralline algae (CCA) bacterial community composition is algal species specific at the taxonomic phylum level. Stacked bar plot visualising the mean relative abundance per family for each CCA species. The most abundant 15 taxa are listed here, and less abundant taxa are grouped in the ‘Other’ category. **Figure S6:** Crustose coralline (CCA) core bacterial communities (75% persistence) are structured by host species. (A) The non‐metric multidimensional scaling (nMDS) plot shows partitioning of samples by CCA species (colour). (B) Stacked bar plot details the relative abundance of each CCA sample at the taxonomic phylum level. The most abundant 15 phyla are listed here with less abundant taxa grouped in the ‘Other’ category. **Figure S7:** Crustose coralline algae (CCA) 100% core bacterial community composition is algal species specific at the taxonomic family level. Stacked bar plot visualising the mean relative abundance per family for each CCA species. The most abundant 30 taxa are listed here, and less abundant taxa are grouped in the ‘Other’ category. **Figure S8:** Crustose coralline algae (CCA) 75% core bacterial community composition is algal species specific at the taxonomic family level. Stacked bar plot visualising the mean relative abundance per family for each CCA species. The most abundant 30 taxa are listed here, and less abundant taxa are grouped in the ‘Other’ category. **Figure S9:** Deviations between CCA species’ entire bacterial community compositions mirror changes in host phylogeny. Nodes and branch labels are coloured by CCA sub/family and branch labels depict CCA species names. *Ramicrusta* sp. is a non‐CCA species that was used as an outgroup for the host and bacterial community dendrograms. **Figure S10:** Deviations between CCA species’ 100% core bacterial community compositions mirror changes in host phylogeny. Nodes and branch labels are coloured by CCA sub/family and branch labels depict CCA species names. *Ramicrusta* sp. is a non‐CCA species that was used as an outgroup for the host and bacterial community dendrograms. **Table S1:** Crustose coralline algae (CCA) identification, collection site, habitat, and sequence reference information. Light exposure definitions were observational and were determine by the depth and what habitat they were found in. Herbarium reference numbers are from Diaz‐Pulido's personal collection at Griffiths University, Brisbane, Australia. **Table S2:** 16S rRNA amplicon read counts per CCA sample. Three samples were removed due to low read count and are indicated with an asterisk. **Table S3:** Kruskal‐Wallis one‐way variance test statistics of crustose coralline algae (CCA) bacterial community diversity indices. **Table S4:** PERMANOVA variance test statistics comparing the crustose coralline algae (CCA) bacterial communities of groups of CCA species, CCA family, CCA habitat light exposure, and collection site. **Table S5:** ANOVA variance test statistics comparing the dispersion between the crustose coralline algae (CCA) bacterial communities of groups of CCA species, CCA family, CCA habitat light exposure, and collection site. **Table S6:** Amplicon sequence variance (ASV) counts conserved in the core bacterial communities for each crustose coralline algae (CCA) species. **Table S7:** Wilcox pairwise variance test statistics comparing the differing abundances of taxonomic phyla across crustose coralline algae (CCA).

## Data Availability

The bacterial dataset analysed during the current study is available in the NCBI Sequence Read Archive (SRA) (https://www.ncbi.nlm.nih.gov/sra) under the BioProject accession numbers PRJNA1100296. The CCA samples used for molecular identification and phylogenetic trees is available in GenBank under the accession numbers ranging from OP830444 – OP830473.
