# Supplementary material for: Bacterial Communities Associated With Crustose Coralline Algae Are Host‐Specific
Source: Microbiologyopen. 2026 Jan 14;15(1):e70213. doi: 10.1002/mbo3.70213 (PMC12805225; doi:10.1002/mbo3.70213)
Supplement: Supplementary file 1 — Figure S1: Map of crustose coralline algae (CCA) collection sites across Havannah Island and Davies Reef, central Great Barrier Reef, off the coast of Townsville, Australia. Davies Reef represents a low‐turbidity, pristine mid‐shelf reef and Havannah Island represents a fringing reef on an inshore island. Figure S2: CCA samples rarefaction curve. Samples that were removed from low read count are labelled with purple stars, and the sample used as the cutoff to rarify the dataset for alpha diversity analysis is labelled with a green star. Figure S3: Crustose coralline algae (CCA) phylogenetic tree based on Maximum Likelihood trimmed to the CCA vouchers used in this study of combined psbA and rbcL concatenated sequences. CCA are shaded in their shared sub/family and values at the beginning of branches represent maximum likelihood bootstrap values (%). Reference numbers located at the beginning of each CCA name are the herbarium numbers used in the personal collection of Diaz‐Pulido at Griffith University, Brisbane, Australia. Figure S4: Crustose coralline algae (CCA) bacterial communities vary depending on (A) CCA family, (B) CCA habitat light exposure, and (C) collection site. Each point represents a CCA sample and nMDS clusters used Bray‐Curtis distance on log transformed amplicon sequence variant (ASV) counts. Figure S5: Crustose coralline algae (CCA) bacterial community composition is algal species specific at the taxonomic phylum level. Stacked bar plot visualising the mean relative abundance per family for each CCA species. The most abundant 15 taxa are listed here, and less abundant taxa are grouped in the ‘Other’ category. Figure S6: Crustose coralline (CCA) core bacterial communities (75% persistence) are structured by host species. (A) The non‐metric multidimensional scaling (nMDS) plot shows partitioning of samples by CCA species (colour). (B) Stacked bar plot details the relative abundance of each CCA sample at the taxonomic phylum level. The most abundant [file MBO3-15-e70213-s001.docx]

**Supplementary Information**


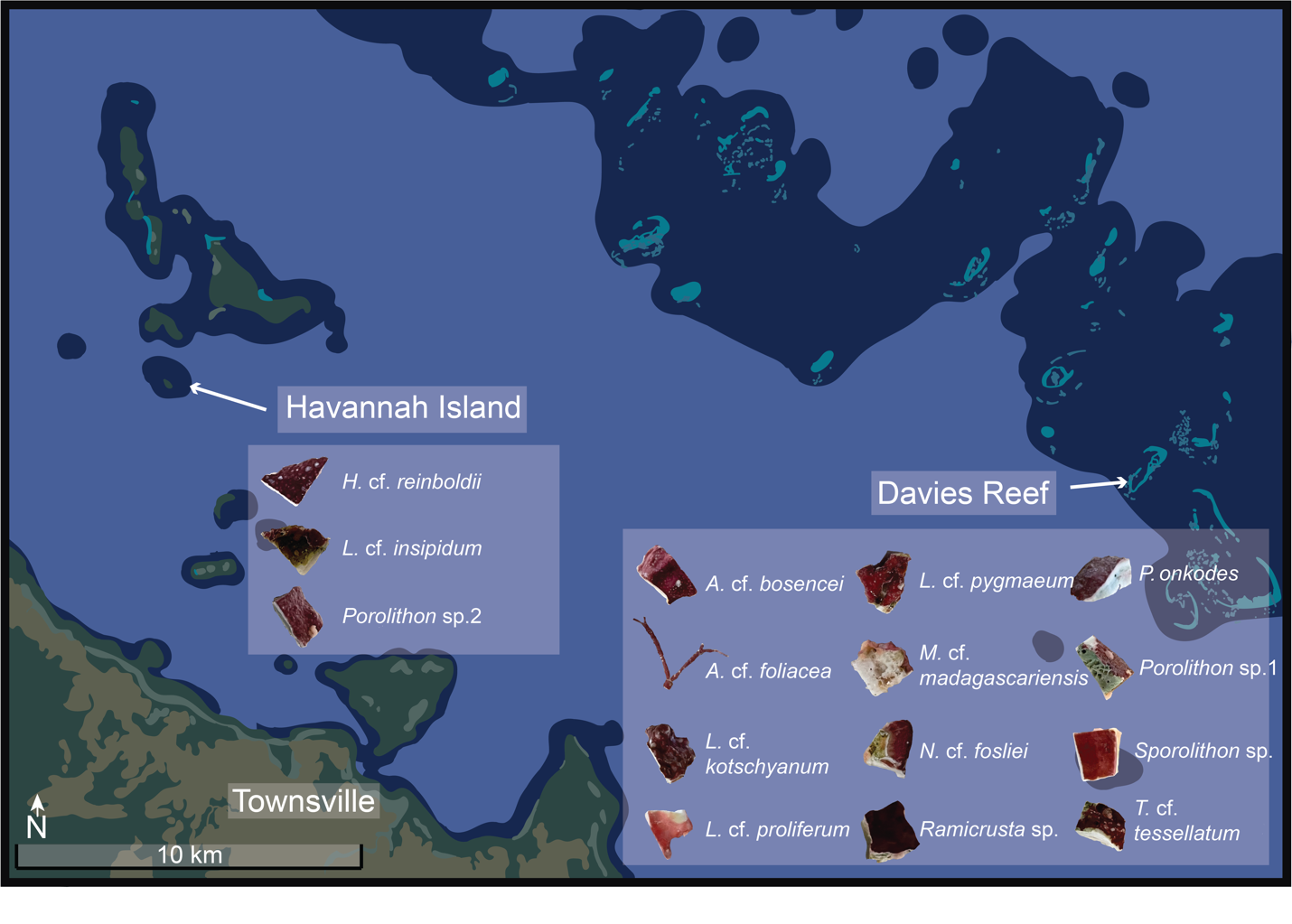


Figure S1. Map of crustose coralline algae (CCA) collection sites across Havannah Island and Davies Reef, central Great Barrier Reef, off the coast of Townsville, Australia. Davies Reef represents a low-turbidity, pristine mid-shelf reef and Havannah Island represents a fringing reef on an inshore island.


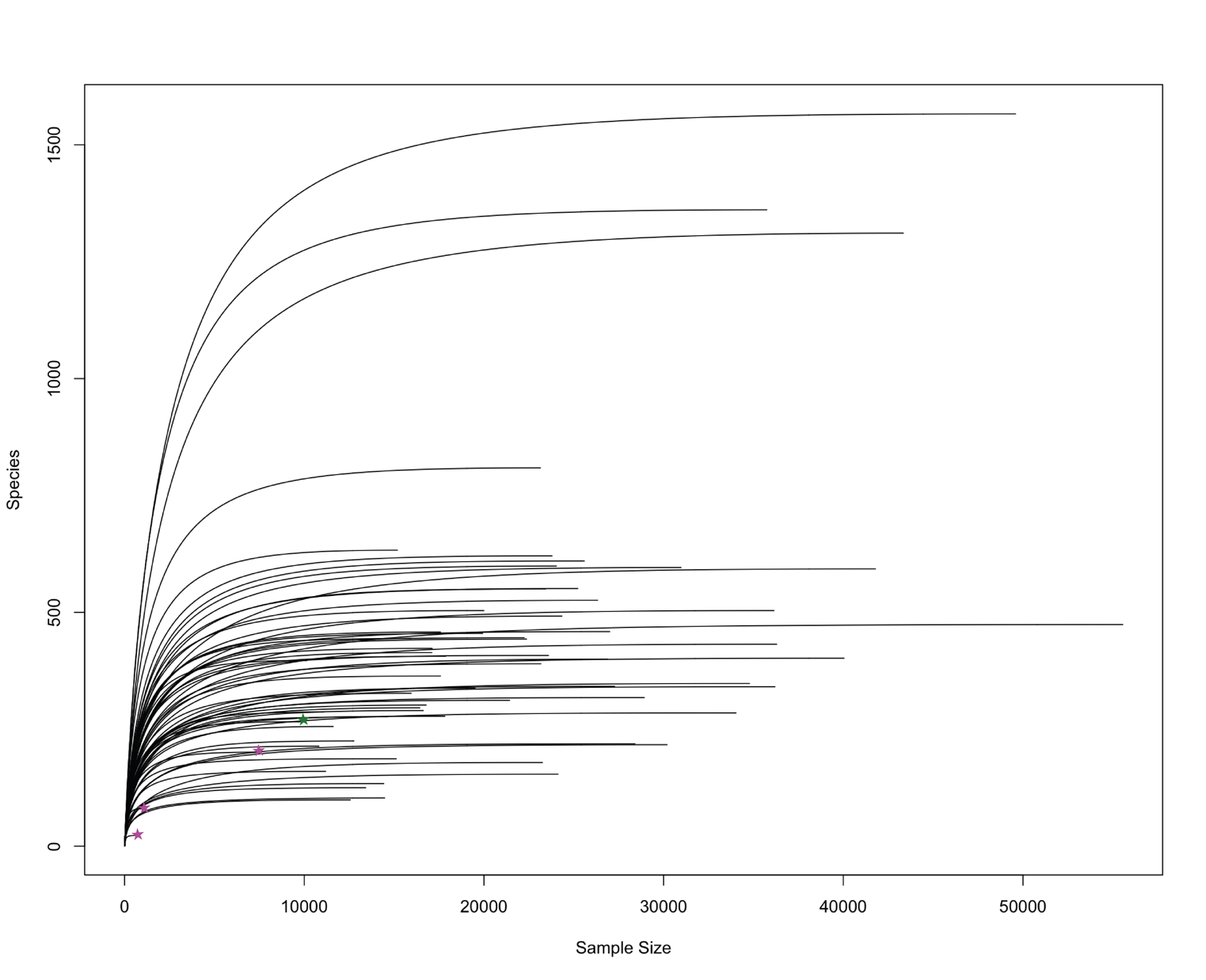


Figure S2. CCA samples rarefaction curve. Samples that were removed from low read count are labelled with purple stars, and the sample used as the cutoff to rarify the dataset for alpha diversity analysis is labelled with a green star.


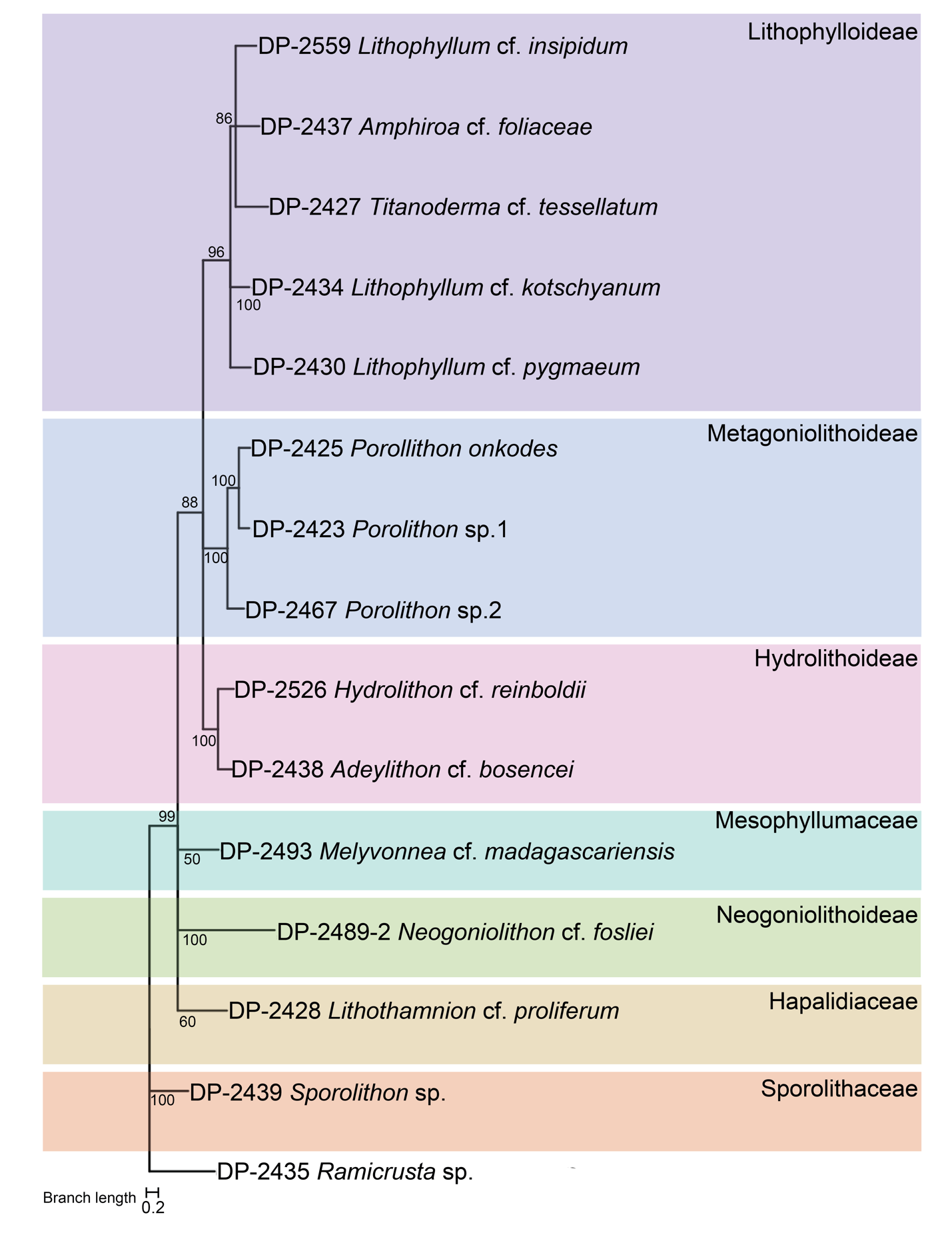


Figure S3: Crustose coralline algae (CCA) phylogenetic tree based on Maximum Likelihood trimmed to the CCA vouchers used in this study of combined *psb*A and *rbc*L concatenated sequences. CCA are shaded in their shared sub/family and values at the beginning of branches represent maximum likelihood bootstrap values (%). Reference numbers located at the beginning of each CCA name are the herbarium numbers used in the personal collection of Diaz-Pulido at Griffith University, Brisbane, Australia.


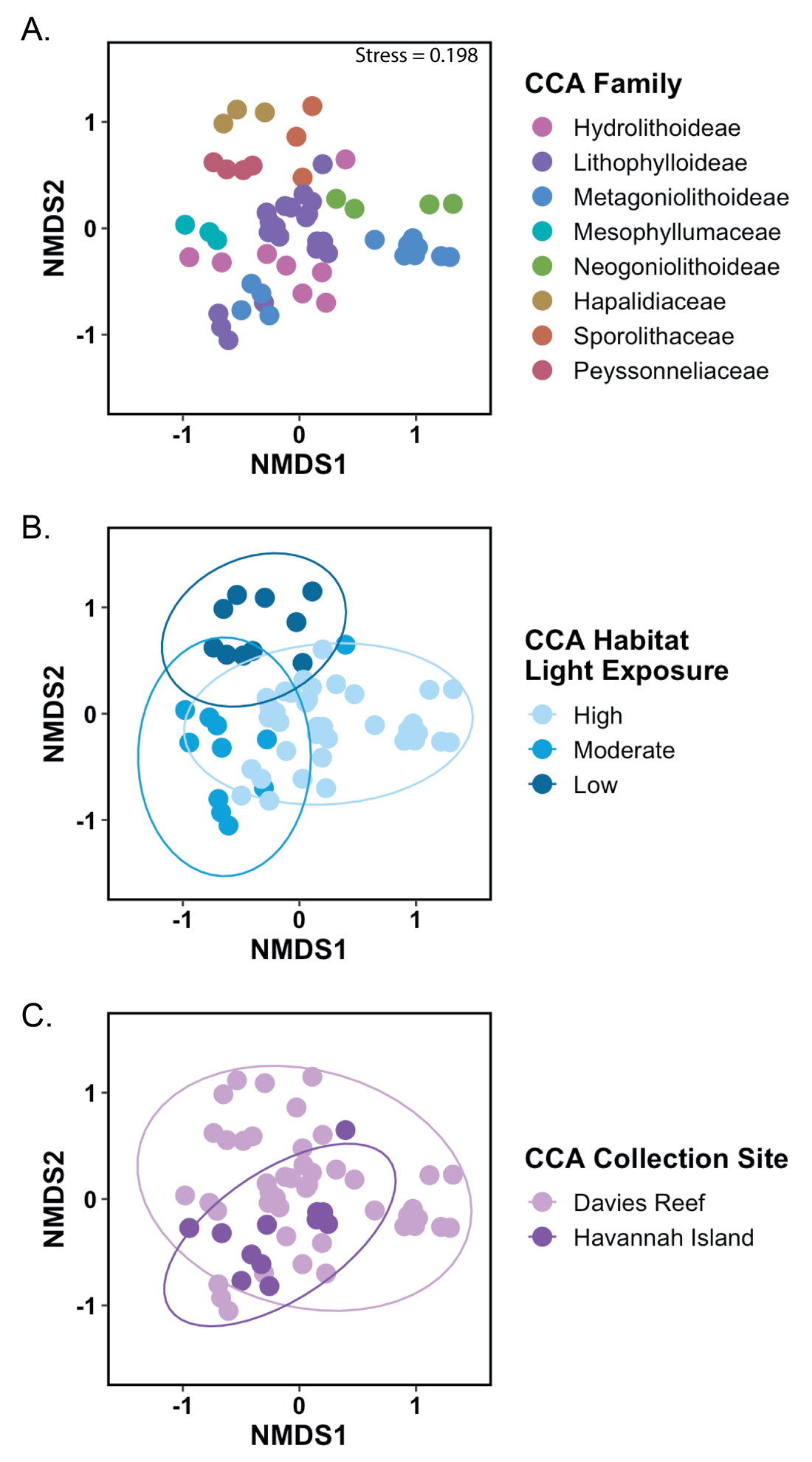


Figure S4: Crustose coralline algae (CCA) bacterial communities vary depending on (A) CCA family, (B) CCA habitat light exposure, and (C) collection site. Each point represents a CCA sample and nMDS clusters used Bray-Curtis distance on log transformed amplicon sequence variant (ASV) counts.


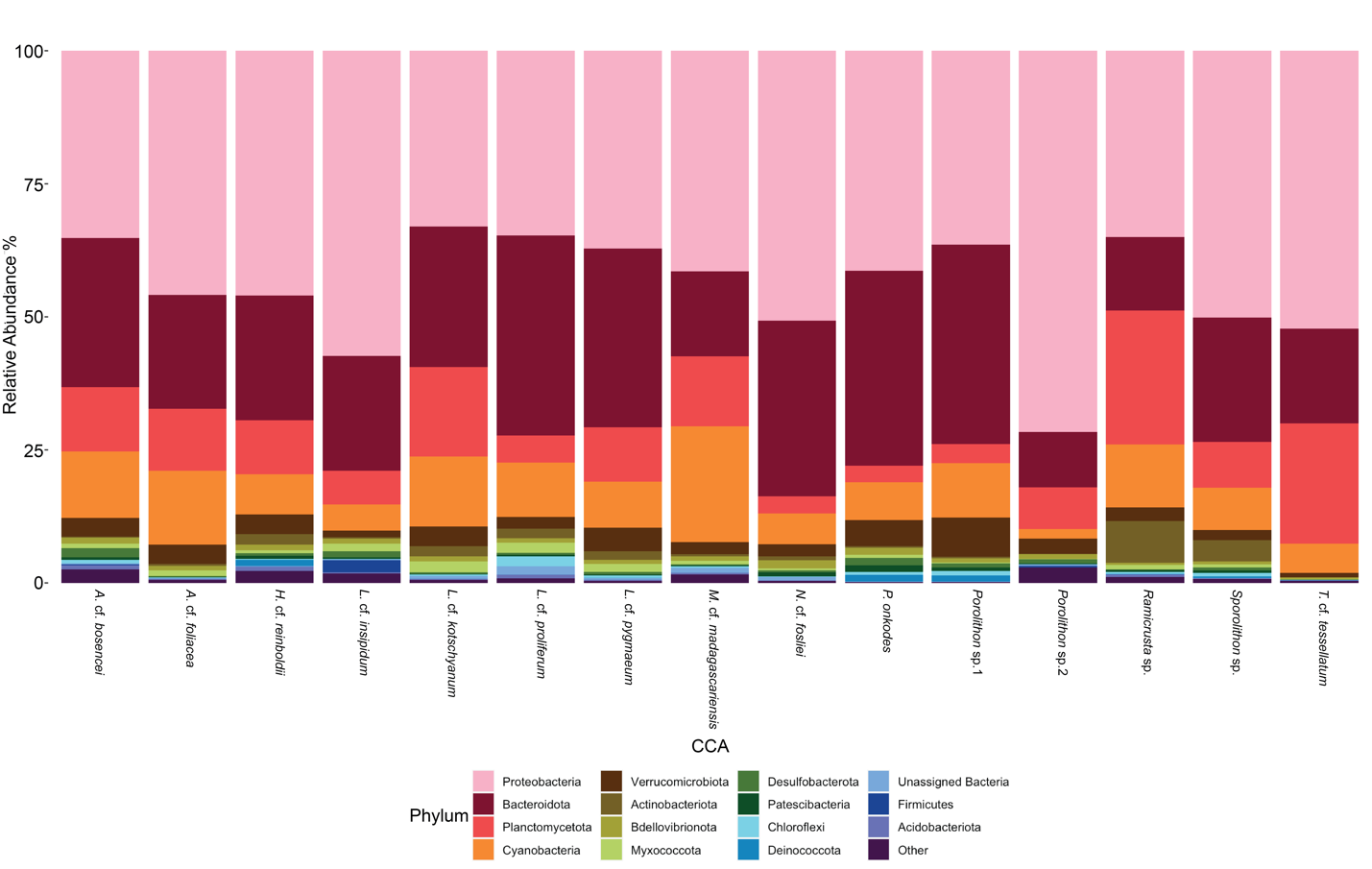


Figure S5. Crustose coralline algae (CCA) bacterial community composition is algal species specific at the taxonomic phylum level. Stacked bar plot visualising the mean relative abundance per family for each CCA species. The most abundant 15 taxa are listed here, and less abundant taxa are grouped in the ‘Other’ category.


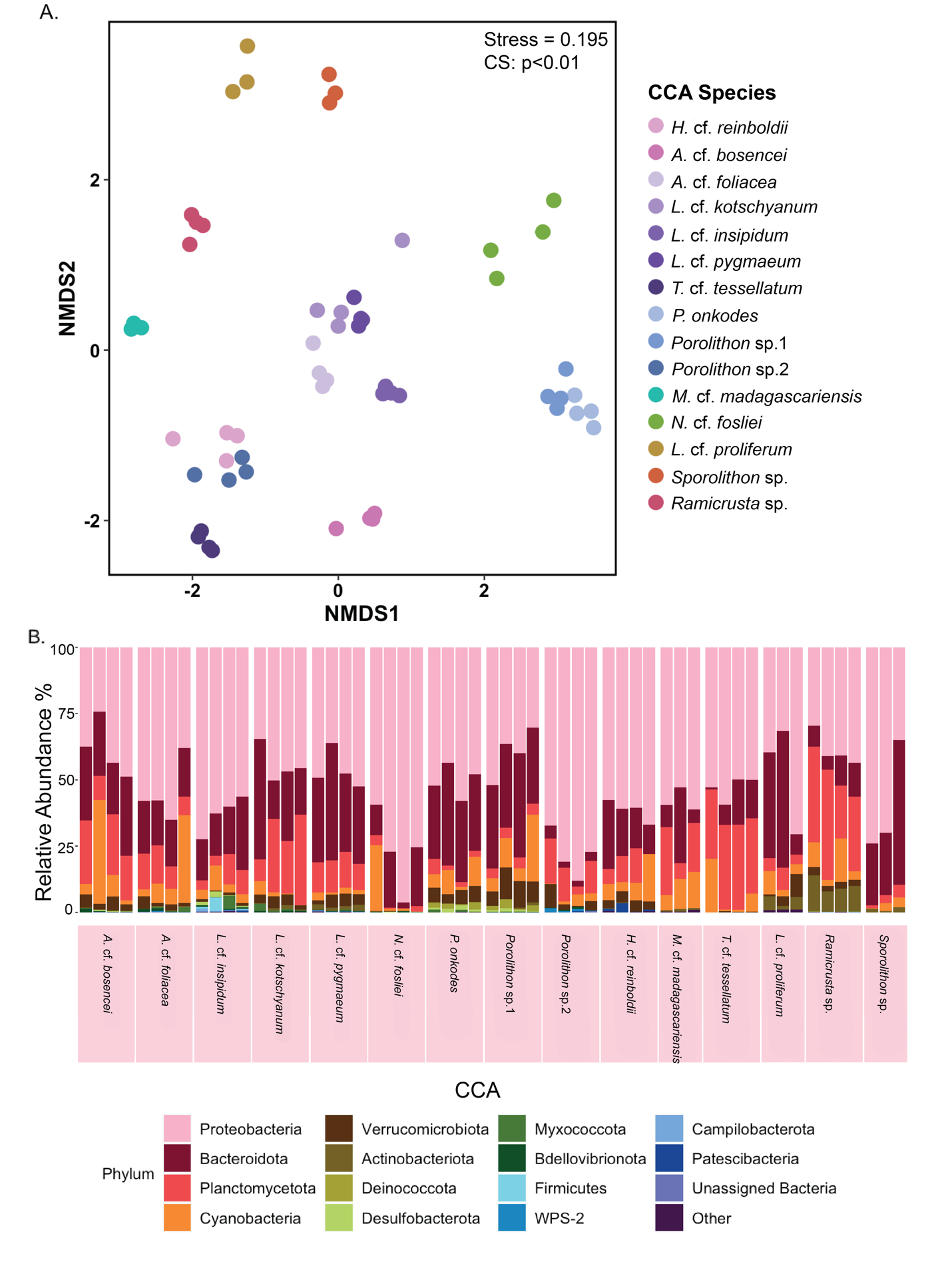


Figure S6. Crustose coralline (CCA) core bacterial communities (75% persistence) are structured by host species. (A) The non-metric multidimensional scaling (nMDS) plot shows partitioning of samples by CCA species (colour). (B) Stacked bar plot details the relative abundance of each CCA sample at the taxonomic phylum level. The most abundant 15 phyla are listed here with less abundant taxa grouped in the ‘Other’ category.


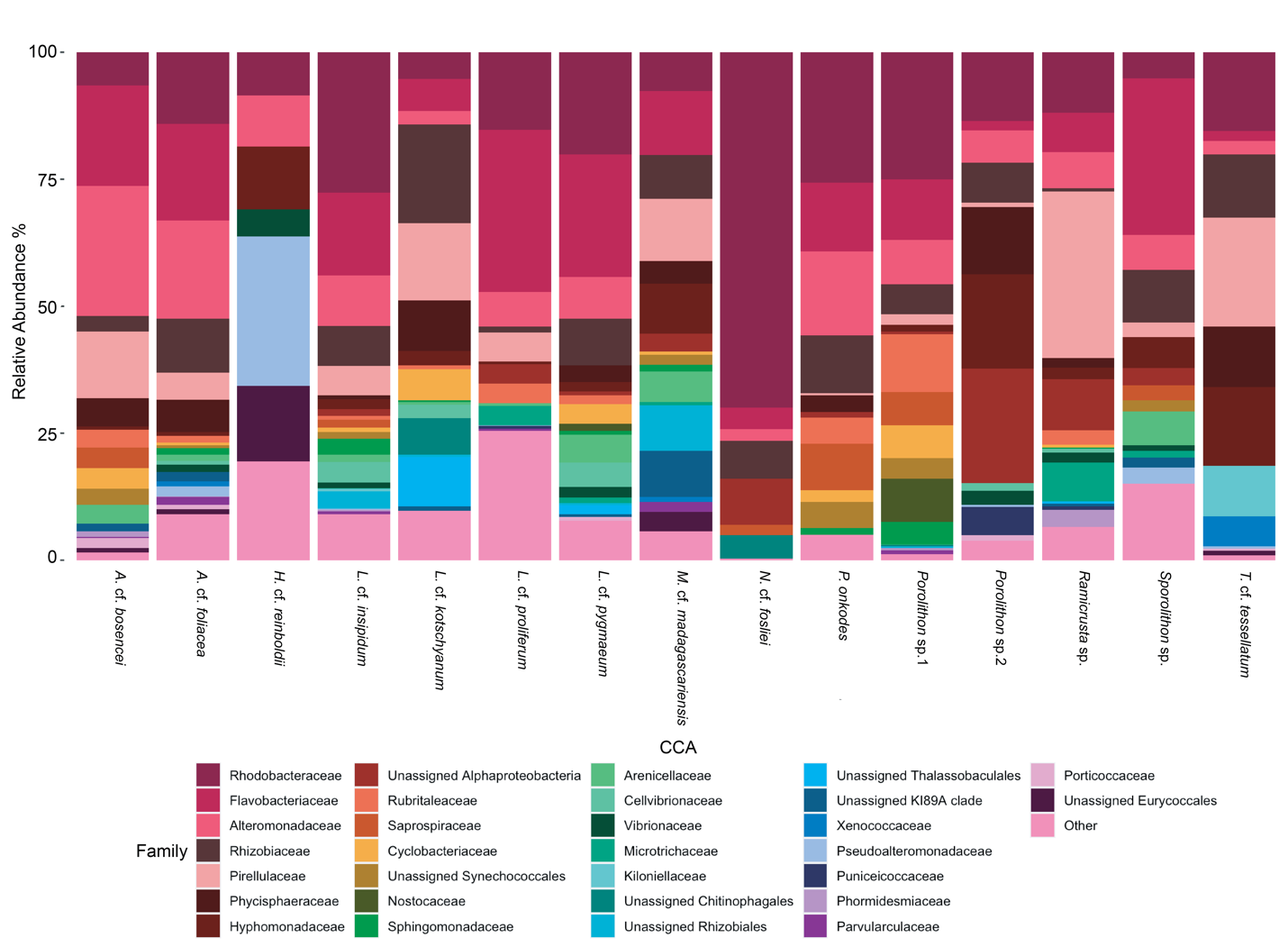


Figure S7. Crustose coralline algae (CCA) 100% core bacterial community composition is algal species specific at the taxonomic family level. Stacked bar plot visualising the mean relative abundance per family for each CCA species. The most abundant 30 taxa are listed here, and less abundant taxa are grouped in the ‘Other’ category.


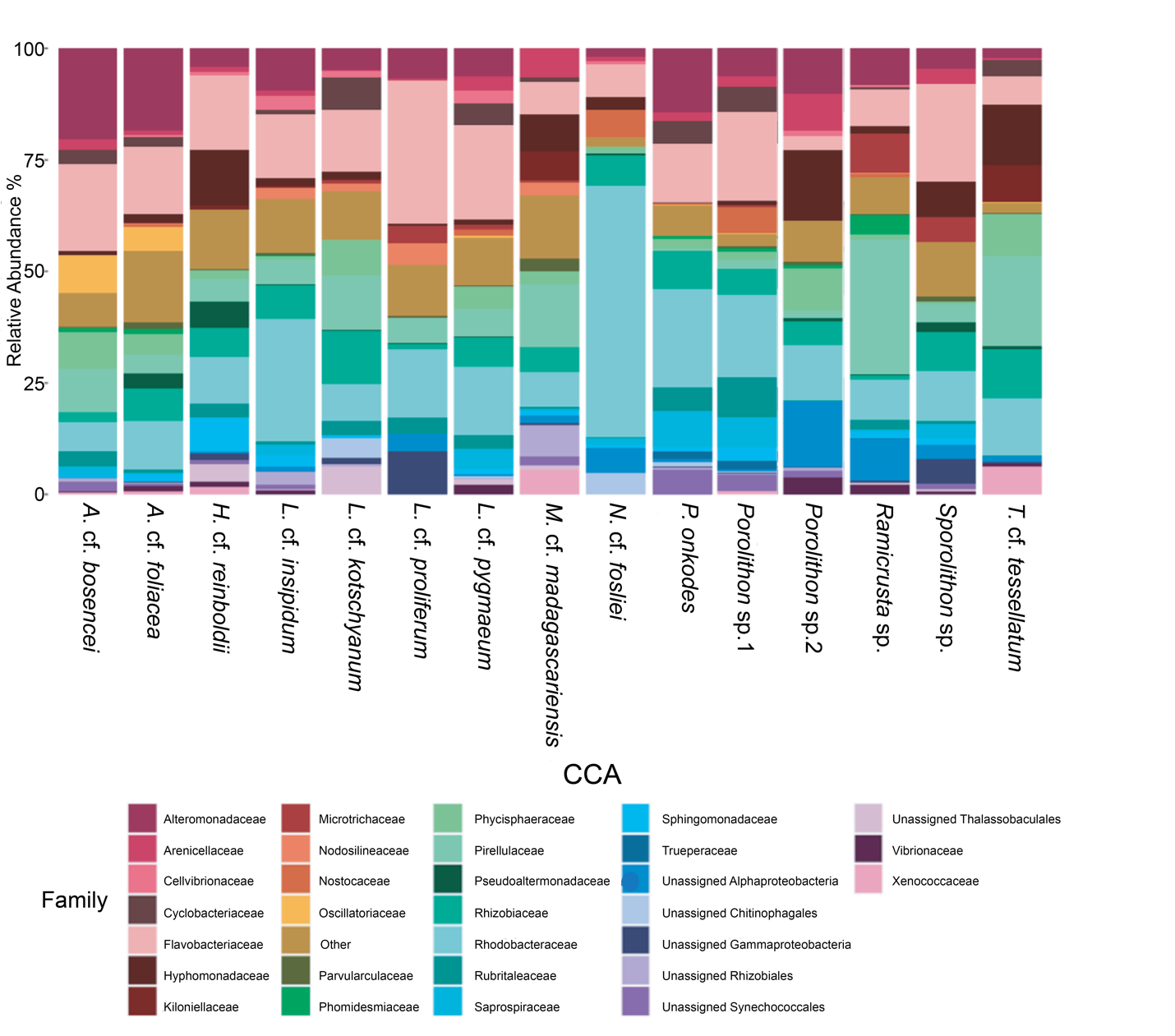


Figure S8. Crustose coralline algae (CCA) 75% core bacterial community composition is algal species specific at the taxonomic family level. Stacked bar plot visualising the mean relative abundance per family for each CCA species. The most abundant 30 taxa are listed here, and less abundant taxa are grouped in the ‘Other’ category.


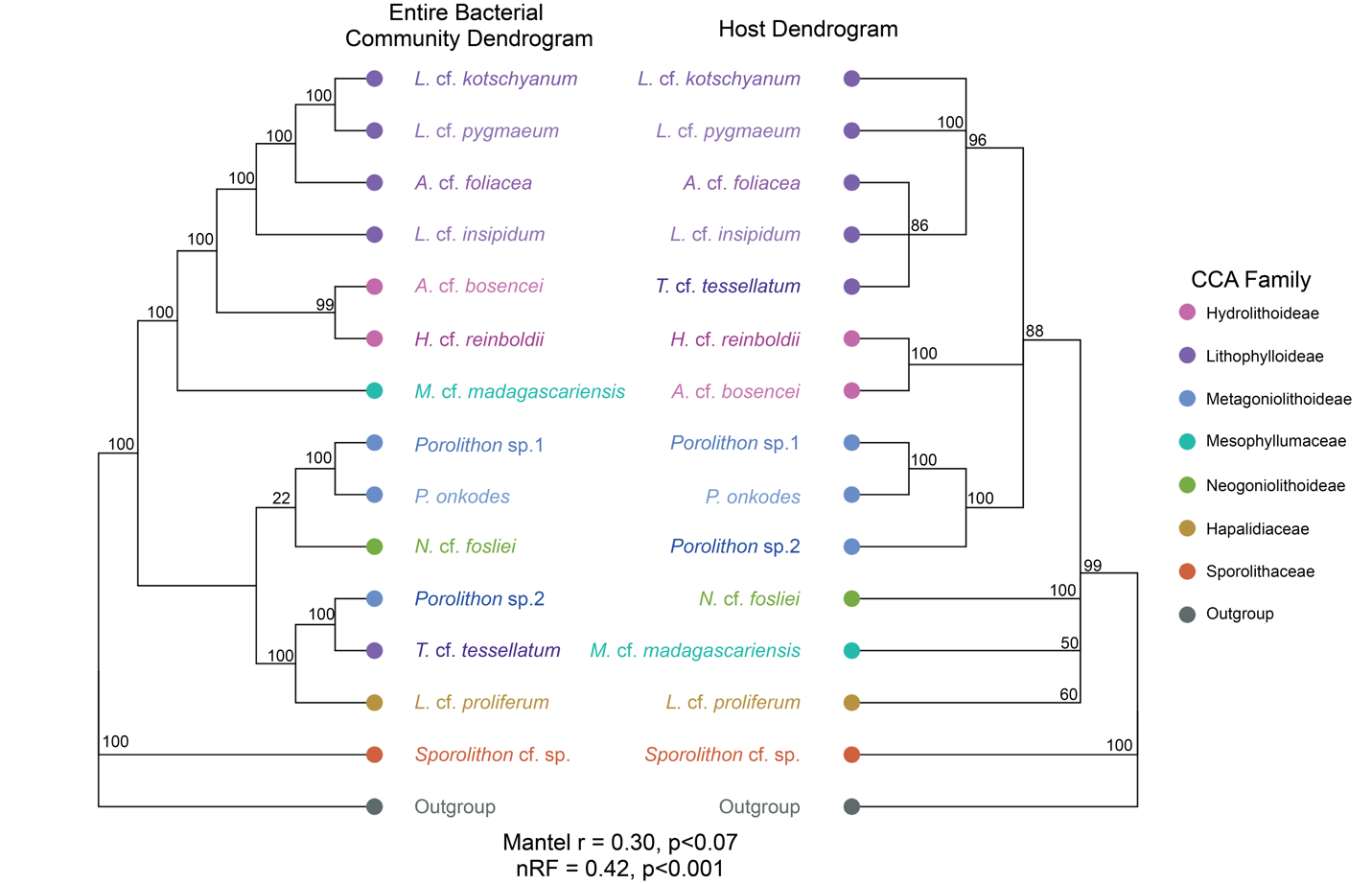


Figure S9. Deviations between CCA species’ entire bacterial community compositions mirror changes in host phylogeny. Nodes and branch labels are coloured by CCA sub/family and branch labels depict CCA species names. *Ramicrusta* sp. is a non-CCA species that was used as an outgroup for the host and bacterial community dendrograms.


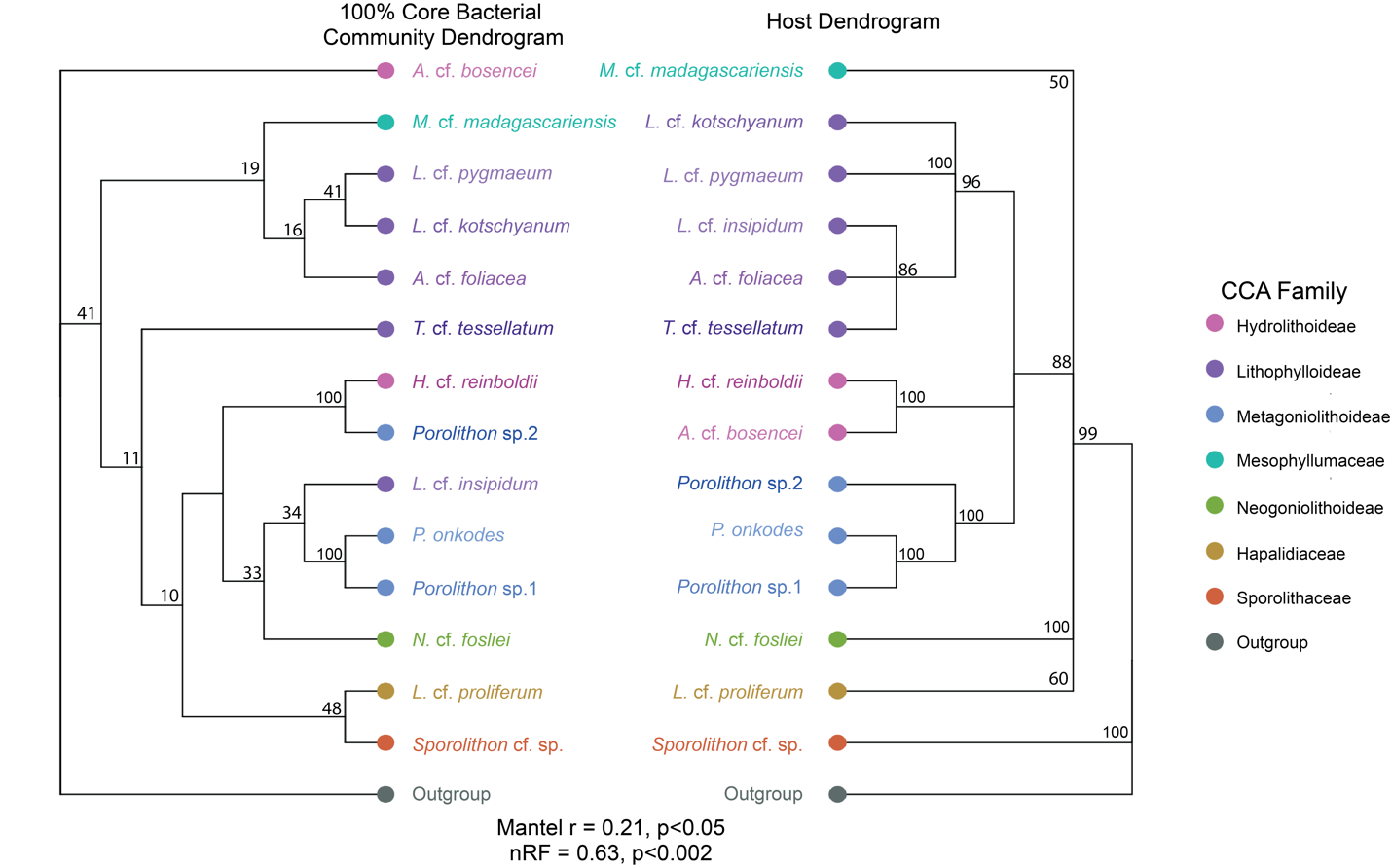


Figure S10. Deviations between CCA species’ 100% core bacterial community compositions mirror changes in host phylogeny. Nodes and branch labels are coloured by CCA sub/family and branch labels depict CCA species names. *Ramicrusta* sp. is a non-CCA species that was used as an outgroup for the host and bacterial community dendrograms.

Table S1. Crustose coralline algae (CCA) identification, collection site, habitat, and sequence reference information. Light exposure definitions were observational and were determine by the depth and what habitat they were found in. Herbarium reference numbers are from Diaz-Pulido’s personal collection at Griffiths University, Brisbane, Australia.

| CCA Species | Acronym | CCA Family/Sub family | Collection site | Sampling Depth (m) | Habitat | Light Exposure | Herbarium numbers | GenBank | |
| --- | --- | --- | --- | --- | --- | --- | --- | --- | --- |
|  |  |  |  |  |  |  |  | psbA | rbcL |
| *Adeylithon* cf. *bosencei* | Abos | Hydrolithoideae | Davies Reef | 2-4 | Shallow – Deep reef | High | DP-2438 | OP830454 | OP830469 |
| *Amphiroa* cf.  *foliacea* | Afol | Lithophylloideae | Davies Reef | 4 | Shallow – Mid reef | High | DP-2437 | OP830453 | OP830468 |
| *Hydrolithon* cf. *reinboldii* | Hrei | Hydrolithoideae | Havannah Island | 2-3 | Shallow – Deep reef | Moderate | DP-2526 | OP830457 | OP830472 |
| *Lithophyllum* cf. *insipidium* | Lins | Lithophylloideae | Havannah Island | 2-3 | Crest, Shallow reef | High | DP-2559 | OP830456 | OP830471 |
| *Lithophyllum* cf. *kotschyanum* | Lkot | Lithophylloideae | Davies Reef | 4 | Reef Crest | High | DP-2434 | OP830451 | OP830466 |
| *Lithothamnion* cf.  *proliferum* | Lpro | Hapalidiaceae | Davies Reef | 6 | Crevices, Caves | Low | DP-2428 | OP830448 | OP830463 |
| *Lithophyllum* cf. *pygmaeum* | Lpyg | Lithophylloideae | Davies Reef | 4 | Crest, Shallow reef | High | DP-2430 | OP830449 | OP830464 |
| *Melyvonnea* cf. *madagascariensis* | Mmad | Mesophyllumaceae | Davies Reef | 4-5 | Shallow – Deep reef | Moderate | DP-2439 | OP830542 | OP830467 |
| *Neogoniolithon* cf. *fosliei* | Nfos | Neogoniolithoideae | Davies Reef | 3 | Reef Crest | High | DP-2489-2 | OP830540 | OP830465 |
| *Ramicrusta* sp. | Pey | Peyssonneliaceae | Davies Reef | 4 | Crevices, Caves | Low | DP-2435 | OP830458 | OP830473 |
| *Porolithon onkodes* | Ponk | Metagoniolithoideae | Davies Reef | 2 | Reef Crest | High | DP-2425 | OP830445 | OP830460 |
| *Porolithon* sp.1 | P_sp1 | Metagoniolithoideae | Davies Reef | 2 | Reef Crest | High | DP-2423 | OP830444 | OP830460 |
| *Porolithon* sp.2 | P_sp2 | Metagoniolithoideae | Havannah Island | 3 | Reef Crest | High | DP-2467 | OP830446 | OP830461 |
| *Sporolithon sp.* | Spo | Sporolithaceae | Davies Reef | 6 | Crevices, Caves | Low | DP-2439 | OP830455 | OP830470 |
| *Titanoderma* cf. *tessellatum* | Ttes | Lithophylloideae | Davies Reef | 4 | Shallow – Deep reef | Moderate | DP-2427 | OP830447 | OP830462 |

Table S2. 16S rRNA amplicon read counts per CCA sample. Three samples were removed due to low read count and are indicated with an asterisk.

| CCA | Sample Label | 16S rRNA reads | Removed Samples |
| --- | --- | --- | --- |
| *Adeylithon* cf. *bosencei* | SF0940 | 25980 |  |
|  | SF0941 | 22672 |  |
|  | SF0942 | 21593 |  |
|  | SF0943 | 20224 |  |
| *Amphiroa* cf. *foliacea* | SF0939 | 24368 |  |
|  | SF0936 | 24313 |  |
|  | SF0937 | 23364 |  |
|  | SF0938 | 18408 |  |
| *Hydrolithon* cf. *reinboldii* | SF0966 | 18877 |  |
|  | SF0967 | 18614 |  |
|  | SF0964 | 18276 |  |
|  | SF0965 | 10567 |  |
| *Lithophyllum* cf. *insipidium* | SF0959 | 45854 |  |
|  | SF0957 | 28180 |  |
|  | SF0956 | 25422 |  |
|  | SF0958 | 18784 |  |
| *Lithophyllum* cf. *kotschyanum* | SF0950 | 33182 |  |
|  | SF0951 | 27426 |  |
|  | SF0949 | 25678 |  |
|  | SF0948 | 13700 |  |
| *Lithophyllum* cf. *pygmaeum* | SF0909 | 28560 |  |
|  | SF0910 | 28351 |  |
|  | SF0908 | 25661 |  |
|  | SF0911 | 25364 |  |
| *Lithothamnion* cf. *proliferum* | SF0912 | 14630 |  |
|  | SF0915 | 11559 |  |
|  | SF0913 | 11448 |  |
|  | SF0914 | 602 | * |
| *Melyvonnea* cf. *madagascariensis* | SF0928 | 17621 |  |
|  | SF0930 | 17038 |  |
|  | SF0931 | 16962 |  |
|  | SF0929 | 8388 | * |
| *Neogoniolithon* cf. *fosliei* | SF0924 | 39247 |  |
|  | SF0925 | 37571 |  |
|  | SF0926 | 24575 |  |
|  | SF0927 | 16327 |  |
| *Porolithon onkodes* | SF0919 | 38844 |  |
|  | SF0918 | 34415 |  |
|  | SF0916 | 30377 |  |
|  | SF0917 | 16035 |  |
| *Porolithon* sp.1 | SF0923 | 57576 |  |
|  | SF0921 | 41752 |  |
|  | SF0920 | 37072 |  |
|  | SF0922 | 24429 |  |
| *Porolithon* sp.2 | SF0954 | 30580 |  |
|  | SF0952 | 29270 |  |
|  | SF0955 | 27523 |  |
|  | SF0953 | 16726 |  |
| *Ramicrusta sp.* | SF0932 | 27391 |  |
|  | SF0933 | 21180 |  |
|  | SF0935 | 19032 |  |
|  | SF0934 | 17174 |  |
| *Sporolithon sp.* | SF0944 | 17260 |  |
|  | SF0947 | 15161 |  |
|  | SF0945 | 11179 |  |
|  | SF0946 | 1033 | * |
| *Titanoderma* cf. *tessellatum* | SF0906 | 17323 |  |
|  | SF0907 | 16202 |  |
|  | SF0904 | 15642 |  |
|  | SF0905 | 13656 |  |

Table S3. Kruskal-Wallis one-way variance test statistics of crustose coralline algae (CCA) bacterial community diversity indices

| Diversity Test | Chi-squared | Df | p-value |
| --- | --- | --- | --- |
| Observed Richness | 34.448 | 14 | 0.002 |
| Shannon | 35.262 | 14 | 0.001 |

Table S4. PERMANOVA variance test statistics comparing the crustose coralline algae (CCA) bacterial communities of groups of CCA species, CCA family, CCA habitat light exposure, and collection site.

| Source of variation | df | SS | R2 | MS | F Model | p value |
| --- | --- | --- | --- | --- | --- | --- |
| CCA species | 14 | 12.74 | 0.53 | 0.91 | 3.54 | 0.001 |
| Residuals | 44 | 11.31 | 0.47 | 0.26 |  |  |
| Total | 58 | 24.10 | 1 |  |  |  |
|  |  |  |  |  |  |  |
| Source of variation | df | SS | R2 | MS | F Model | p value |
| CCA family | 7 | 6.54 | 0.28 | 0.94 | 2.77 | 0.001 |
| Residuals | 49 | 16.53 | 0.72 | 0.38 |  |  |
| Total | 56 | 23.08 | 1 |  |  |  |
|  |  |  |  |  |  |  |
| Source of variation | df | SS | R2 | MS | F Model | p value |
| CCA habitat light exposure | 2 | 2.20 | 0.10 | 1.10 | 2.85 | 0.001 |
| Residuals | 54 | 20.88 | 0.90 | 0.39 |  |  |
| Total | 56 | 23.08 | 1 |  |  |  |
|  |  |  |  |  |  |  |
| Source of variation | df | SS | R2 | MS | F Model | p value |
| CCA collection site | 1 | 1.02 | 0.04 | 1.02 | 2.54 | 0.001 |
| Residuals | 55 | 22.06 | 0.96 | 0.40 |  |  |
| Total | 56 | 23.08 | 1 |  |  |  |
|  |  |  |  |  |  |  |

Table S5. ANOVA variance test statistics comparing the dispersion between the crustose coralline algae (CCA) bacterial communities of groups of CCA species, CCA family, CCA habitat light exposure, and collection site.

| Response to distances | df | SS | R2 | F value | p value |
| --- | --- | --- | --- | --- | --- |
| CCA species | 14 | 0.20 | 0.014 | 9.03 | 0.001 |
| Residuals | 42 | 0.07 | 0.002 |  |  |

| Response to distances | df | SS | R2 | F value | p value |
| --- | --- | --- | --- | --- | --- |
| CCA family | 7 | 0.56 | 0.008 | 3.16 | 0.008 |
| Residuals | 49 | 0.12 | 0.003 |  |  |
| Response to distances | df | SS | R2 | F value | p value |
| CCA habitat light exposure | 2 | 0.0008 | 0.0004 | 0.33 | 0.7195 |
| Residuals | 54 | 0.07 | 0.0012 |  |  |
| Response to distances | df | SS | R2 | F value | p value |
| CCA collection site | 1 | 0.019 | 0.019 | 13.98 | 0.0004 |
| Residuals | 55 | 0.07 | 0.0013 |  |  |

Table S6. Amplicon sequence variance (ASV) counts conserved in the core bacterial communities for each crustose coralline algae (CCA) species.

| CCA | Number of ASVs 100% persistence & 0.01% relative abundance (100% Core bacterial communities) | Number of ASVs 75% persistence & 0.01% relative abundance (75% Core bacterial communities) | Number of ASVs 0.01% relative abundance | Sample size (n) |
| --- | --- | --- | --- | --- |
| *Adeylithon* cf. *bosencei* | 38 | 85 | 1047 | 4 |
| *Amphiroa* cf. *foliacea* | 64 | 147 | 1385 | 4 |
| *Hydrolithon* cf. *reinboldii* | 12 | 53 | 1324 | 4 |
| *Lithophyllum* cf. *insipidium* | 117 | 207 | 1158 | 4 |
| *Lithophyllum* cf. *kotschyanum* | 47 | 134 | 1544 | 4 |
| *Lithophyllum* cf. *pygmaeum* | 67 | 174 | 1569 | 4 |
| *Lithothamnion* cf. *proliferum* | 35 | 35 | 649 | 3 |
| *Melyvonnea* cf. *madagascariensis* | 39 | 39 | 1151 | 3 |
| *Neogoniolithon* cf. *fosliei* | 12 | 52 | 963 | 4 |
| *Porolithon* *onkodes* | 58 | 133 | 827 | 4 |
| *Porolithon* sp.1 | 71 | 160 | 1151 | 4 |
| *Porolithon* sp.2 | 32 | 73 | 814 | 4 |
| *Ramicrusta* sp. | 60 | 149 | 1455 | 4 |
| *Sporolithon* sp. | 35 | 35 | 723 | 3 |
| *Titanoderma* cf. *tessellatum* | 31 | 55 | 347 | 4 |

Table S7. Wilcox pairwise variance test statistics comparing the differing abundances of taxonomic phyla across crustose coralline algae (CCA)

| Taxa | CCA | *T.* cf. *tessellatum* |  |
| --- | --- | --- | --- |
| Bacteriodota | *A.* cf. *bosencei* | **0.029** |  |
|  | *A.* cf. *foliacea* | **0.029** |  |
|  | *H.* cf. *reinboldii* | 0.200 |  |
|  | *L.* cf. *insipidium* | 0.114 |  |
|  | *L.* cf. *kotschyanum* | 0.057 |  |
|  | *L.* cf. *proliferum* | 0.229 |  |
|  | *L.* cf. *pygmaeum* | **0.029** |  |
|  | *M.* cf. *madagascariensis* | 1.000 |  |
|  | *N.* cf. *fosliei* | 0.486 |  |
|  | *P. onkodes* | **0.029** |  |
|  | *Porolithon* sp.1 | **0.029** |  |
|  | *Porolithon* sp.2 | 0.343 |  |
|  | *Ramicrusta* sp. | 0.886 |  |
|  | *Sporolithon* sp. | 0.057 |  |
|  |  |  |  |
|  |  |  |  |
|  |  | *Porolithon* sp.2 |  |
| Desulfobacterota | *P.* *onkodes* | **0.021** |  |
|  | *Porolithon* sp.1 | **0.021** |  |
|  |  |  |  |
|  |  | *N.* cf. *fosliei* | *T.* cf. *tessellatum* |
| Planctomycetota | *A.* cf. *bosencei* | **0.029** | **0.029** |
|  | *A.* cf. *foliacea* | **0.029** | **0.029** |
|  | *H.* cf. *reinboldii* | 0.343 | **0.029** |
|  | *L.* cf. *insipidium* | 0.057 | **0.029** |
|  | *L.* cf. *kotschyanum* | **0.029** | 0.486 |
|  | *L.* cf. *proliferum* | 0.114 | 0.057 |
|  | *L.* cf. *pygmaeum* | **0.029** | **0.029** |
|  | *M.* cf. *madagascariensis* | **0.029** | **0.029** |
|  | *N.* cf. *fosliei* | **-** | **0.029** |
|  | *P.* *onkodes* | 0.486 | **0.029** |
|  | *Porolithon* sp.1 | 0.057 | **0.029** |
|  | *Porolithon* sp.2 | 0.057 | **0.029** |
|  | *Ramicrusta* sp*.* | **0.029** | 0.886 |
|  | *Sporolithon* sp. | 0.057 | **0.029** |
|  | *T.* cf. *tessellatum* | **0.029** | - |
|  |  |  |  |
|  |  | *T.* cf. *tessellatum* |  |
| Proteobacteria | *A.* cf. *bosencei* | **0.029** |  |
|  | *A.* cf. *foliacea* | 0.686 |  |
|  | *H.* cf. *reinboldii* | 0.057 |  |
|  | *L.* cf. *insipidium* | 0.057 |  |
|  | *L.* cf. *kotschyanum* | 0.114 |  |
|  | *L.* cf. *proliferum* | 0.629 |  |
|  | *L.* cf. *pygmaeum* | 0.114 |  |
|  | *M.* cf. *madagascariensis* | 0.200 |  |
|  | *N.* cf. *fosliei* | **0.029** |  |
|  | *P. onkodes* | 0.486 |  |
|  | *Porolithon* sp.1 | 0.114 |  |
|  | *Porolithon* sp.2 | **0.029** |  |
|  | *Ramicrusta* sp. | **0.029** |  |
|  | *Sporolithon* sp. | 0.114 |  |
|  |  |  |  |
|  |  | *N.* cf *fosliei* | *T.* cf. *tessellatum* |
| Verrumicrobiota | *A.* cf. *bosencei* | **0.029** | **0.029** |
|  | *A.* cf. *foliacea* | **0.029** | **0.029** |
|  | *H.* cf. *reinboldii* | **0.029** | **0.029** |
|  | *L.* cf. *insipidium* | **0.029** | 0.057 |
|  | *L.* cf. *kotschyanum* | **0.029** | **0.029** |
|  | *L.* cf. *proliferum* | 0.057 | 0.057 |
|  | *L.* cf. *pygmaeum* | **0.029** | **0.029** |
|  | *M.* cf. *madagascariensis* | 0.245 | 0.772 |
|  | *N.* cf. *fosliei* | - | 0.384 |
|  | *P. onkodes* | **0.029** | **0.029** |
|  | *Porolithon* sp.1 | **0.029** | **0.029** |
|  | *Porolithon* sp.2 | 0.057 | 0.057 |
|  | *Ramicrusta* sp. | **0.029** | **0.029** |
|  | *Sporolithon* sp*.* | 0.561 | 0.245 |
|  | *T* cf. *tessellatum* | 0.772 | - |

Table S8. Wilcox pairwise variance test statistics comparing the differing abundances of taxonomic families across crustose coralline algae (CCA).

| Taxa | CCA | *N.* cf. *fosliei* |  |
| --- | --- | --- | --- |
| *Rhodobacteraceae* | *A.* cf. *bosencei* | **0.029** |  |
|  | *A.* cf. *foliacea* | **0.029** |  |
|  | *H.* cf. *reinboldii* | **0.029** |  |
|  | *L.* cf. *insipidium* | 0.057 |  |
|  | *L.* cf. *kotschyanum* | **0.029** |  |
|  | *L.* cf. *proliferum* | 0.057 |  |
|  | *L.* cf. *pygmaeum* | **0.029** |  |
|  | *M.* cf. *madagascariensis* | **0.029** |  |
|  | *P. onkodes* | **0.029** |  |
|  | *Porolithon* sp.1 | **0.029** |  |
|  | *Porolithon* sp.2 | **0.029** |  |
|  | *Ramicrusta* sp. | **0.029** |  |
|  | *Sporolithon* sp. | **0.029** |  |
|  | *T.* cf. *tessellatum* | **0.029** |  |
|  |  |  |  |
|  |  | *H.* cf. *reinboldii* |  |
| *Psuedoalteromonadaceae* | *A.* cf. *bosencei* | **0.021** |  |
|  | *A.* cf. *foliacea* | 0.486 |  |
|  | *L.* cf. *insipidium* | **0.029** |  |
|  | *L.* cf. *kotschyanum* | **0.029** |  |
|  | *L.* cf. *proliferum* | 0.057 |  |
|  | *L.* cf. *pygmaeum* | **0.029** |  |
|  | *M.* cf. *madagascariensis* | **0.021** |  |
|  | *N.* cf. *fosliei* | **0.029** |  |
|  | *P.* *onkodes* | **0.021** |  |
|  | *Porolithon* sp.1 | **0.021** |  |
|  | *Porolithon* sp.2 | **0.029** |  |
|  | *Ramicrusta* sp. | **0.029** |  |
|  | *Sporolithon* sp. | **0.029** |  |
|  | *T.* cf. *tessellatum* | **0.029** |  |
|  |  |  |  |
|  |  | *Ramicrusta* sp. | *Sporolithon* sp. |
| *Microtrichaceae* | *A.* cf. *bosencei* | 0.021 | 0.021 |
|  | *A.* cf. *foliacea* | 0.029 | 0.029 |
|  | *H.* cf. *reinboldii* | 0.021 | 0.021 |
|  | *L.* cf. *insipidium* | 0.029 | 0.029 |
|  | *L.* cf. *kotschyanum* | 0.029 | 0.114 |
|  | *L.* cf. *proliferum* | 0.057 | 0.400 |
|  | *L.* cf. *pygmaeum* | 0.029 | 0.200 |
|  | *M.* cf. *madagascariensis* | 0.029 | 0.057 |
|  | *N.* cf. *fosliei* | 0.021 | 0.021 |
|  | *P.* *onkodes* | 0.029 | 0.029 |
|  | *Porolithon* sp.1 | 0.029 | 0.114 |
|  | *Porolithon* sp.2 | 0.021 | 0.021 |
|  | *Ramicrusta* sp. | - | 0.343 |
|  | *Sporolithon* sp. | 0.343 | - |
|  | *T.* cf. *tessellatum* | 0.021 | 0.021 |
|  |  |  |  |
|  |  | *Porolithon* sp.2 |  |
| *Vibrionaceae* | *A.* cf. *bosencei* | 0.200 |  |
|  | *A.* cf. *foliacea* | 0.686 |  |
|  | *H.* cf. *reinboldii* | 1.000 |  |
|  | *L.* cf. *insipidium* | 0.886 |  |
|  | *L.* cf. *kotschyanum* | **0.021** |  |
|  | *L.* cf. *proliferum* | **0.044** |  |
|  | *L.* cf. *pygmaeum* | 1.000 |  |
|  | *M.* cf. *madagascariensis* | **0.021** |  |
|  | *N.* cf. *fosliei* | **0.021** |  |
|  | *P.* *onkodes* | **0.021** |  |
|  | *Porolithon* sp.1 | **0.021** |  |
|  | *Ramicrusta* sp*.* | 1.000 |  |
|  | *Sporolithon* sp. | 0.343 |  |
|  | *T.* cf. *tessellatum* | 0.686 |  |
